# Mindfulness, music, visual occlusion in ketamine therapy for depression: do they change outcomes? A qualitative and quantitative analysis of a randomized controlled trial

**DOI:** 10.3389/fpsyt.2025.1642025

**Published:** 2025-09-02

**Authors:** Mina Kheirkhah, Nastasia McDonald, Julia Aepfelbacher, Manivel L. Rengasamy, Sharvari Shivanekar, Crystal Spotts, Iya Cooper, Andrew Baumeister, Elizabeth Bell, Kevin Do-Nguyen, Mary L. Woody, Shabnam Hossein, Ioline D. Henter, Allison C. Nugent, Nadia S. Hejazi, Hamidreza Jamalabadi, Mani Yavi, Martin Walter, Carlos A. Zarate, Rebecca B. Price

**Affiliations:** ^1^ Experimental Therapeutics and Pathophysiology Branch, Intramural Research Program, National Institute of Mental Health, National Institutes of Health, Bethesda, MD, United States; ^2^ Department of Psychiatry and Psychotherapy, Jena University Hospital, Jena, Germany; ^3^ Department of Psychiatry, University of Pittsburgh, Pittsburgh, PA, United States; ^4^ Magnetoencephalography Core, National Institute of Mental Health, Bethesda, MD, United States; ^5^ Department of Psychiatry and Psychotherapy, Philipps University of Marburg, Marburg, Germany

**Keywords:** depression, depression rating scales, eye mask, ketamine, mindfulness, music, qualitative analyses, quantitative analyses

## Abstract

**Introduction:**

This is the first randomized controlled trial to use both qualitative and quantitative methods to evaluate the effects of a combined sensory intervention that included mindfulness, music, and a light-occluding eye mask during antidepressant-dose ketamine treatment for depression.

**Methods:**

Forty-three participants with unipolar depressive disorder enrolled in the study; 22 individuals were randomly assigned to receive mindfulness, music, and eye mask during ketamine infusion, and 21 individuals in the control group received only ketamine without additional interventions. Quantitative analyses assessed the impact of combined sensory intervention on ketamine’s antidepressant effects, and qualitative analyses explored the participants’ experiences.

**Results:**

Depression scores improved significantly and similarly across both groups. However, adding combined sensory interventions to ketamine infusion enriched subjective experience. More participants in the combined sensory intervention group reported deeper engagement, a stronger sense of connection to reality, increased focus on the experience rather than the strangeness of it, moments of relief from sadness, and feelings of awe and spiritual insight compared to the control group. Four individuals in the combined sensory intervention group also reported discomfort.

**Discussion:**

Ketamine’s antidepressant effects remained consistent with or without combined sensory intervention; however, mindfulness, music, and eye mask made the experience more meaningful and emotionally rich for many, though it also introduced discomfort for a few—this outcome might be avoided by making these interventions optional. Given the limited research on combining ketamine with sensory interventions, these results contribute valuable insights and underscore the need for further studies to explore this combined therapeutic approach.

**Clinical trial registration:**

https://clinicaltrials.gov/study/NCT05168735, identifier NCT05168735.

## 1 Introduction

Subanesthetic-dose racemic (*R,S*)-ketamine (hereafter referred to as ketamine) is a promising treatment for depression (1–3). The exponential growth in its utilization reflects a shifting landscape in psychiatric care (4, 5). Nevertheless, some individuals receiving antidepressant-dose ketamine report peculiar experiences, including dissociative symptoms—defined as a disconnect between thoughts, emotions, sensations, and the surrounding environment (6–8), altered perceptions of color, and feelings of floating (9, 10). In this context, clinics are actively embracing diverse strategies to optimize the therapeutic potential of ketamine by cultivating an environment that is both positive and supportive for patients (7, 11–13). Strategies include incorporating familial presence, guidance of supportive staff, providing eye masks to reduce light sensitivity, and allowing patients to recline, fostering a relaxed atmosphere to alleviate potential confusion, agitation, or anxiety during ketamine infusion (11). Given the patient-specific nature of these interventions, a crucial element is aligning strategies with individual preferences to tailor each session to unique needs and comfort levels (11, 13–15).

Amongst these strategies, the integration of music and mindfulness during ketamine infusion therapy is a relatively unexplored area. The existing literature on this topic is sparse and leaves a critical gap in understanding the impact of these interventions on the overall ketamine experience, particularly in the context of treating depression (16, 17). Existing studies suggest that combining music or mindfulness with ketamine/esketamine (the *S*-enantiomer of ketamine) treatments generally reduced distress, provided relaxation, reduced ambient noise, and connected individuals to reality (6, 7, 18–21). Nevertheless, significant variations in individual experiences have been noted; with regard to music, for instance, some studies suggested that individuals found comfort in specific music genres (16, 22), while others felt that music had unpredictable and occasionally negative effects (6). Moreover, a recent randomized, controlled trial investigated the impact of ketamine combined with music and a mindfulness-supportive control condition compared to ketamine alone in individuals with treatment-resistant depression and found that while depressive symptoms were significantly improved in both groups, there were no significant differences between conditions (23). Importantly, the mindfulness component was not standardized and was primarily intended to support relaxation rather than serve as a structured therapeutic intervention.

From a neurobiological standpoint, ketamine rapidly enhances synaptic plasticity by modulating glutamatergic signaling—activating the mechanistic target of rapamycin (mTOR) pathway and increasing brain-derived neurotrophic factor (BDNF) expression—creating a transient window of heightened neural receptivity (e.g., increased connectivity and circuit remodeling) (24, 25). This enhanced plasticity may allow external inputs (such as music and mindfulness) to shape therapeutic effects during infusion. Likewise, psychological models of emotion regulation propose that mindfulness reduces rumination and enhances attention control via neurobiological changes in networks like the insula, cingulate cortex, and prefrontal areas (26). In this context, music therapy during ketamine sessions has been demonstrated to decrease distress and support emotional grounding, facilitating more tolerable experiences (6, 16). Together, these interventions leverage neurobiological and psychological mechanisms, including synaptic plasticity, emotion regulation circuitry, and sensory grounding, to potentially enhance and guide the acute ketamine experience as well as its downstream antidepressant outcomes. Within this framework, the study hypothesis was that adding mindfulness and music would synergistically amplify ketamine-induced synaptic plasticity and emotion regulation, leading to greater reductions in depressive symptom scores and more positive subjective experiences versus ketamine alone.

In addition to music and mindfulness, visual occlusion using eye masks has emerged as a potentially meaningful component of the ketamine experience. Although often used informally to promote relaxation or reduce external distractions, recent findings suggest that eye mask use may alter neural and phenomenological responses during ketamine administration. For example, Farnes and colleagues demonstrated that ketamine infusions administered with versus without eye masks produced distinct profiles of electroencephalogram (EEG) signal diversity and subjective experiences, as measured by the 11-Dimensional Altered States of Consciousness (11D-ASC) scale (27). Specifically, they found that visual occlusion (eyes closed or masked) was associated with stronger correlations between EEG complexity and imagery-related experiences, while eyes-open conditions were linked to higher levels of anxiety and reduced experiences of unity. These findings suggest that light occlusion may influence both the emotional and perceptual dimensions of the ketamine experience and could play an important role in shaping therapeutic outcomes. Despite its routine use in some clinical and psychedelic contexts, eye mask use remains an underexplored variable in ketamine research and warrants further investigation.

To our knowledge, no prior randomized, controlled study has experimentally manipulated the combination of mindfulness, music, and eye mask during ketamine infusion to assess their impact on depressive symptoms using both qualitative and quantitative measures. This study sought to explore the impact of this combined sensory intervention on ketamine therapy for unipolar depressive disorders (primarily major depressive disorder). Forty-three individuals with clinical depression were randomized to receive ketamine infusion plus either a combined mindfulness, music, and eye mask intervention (n=22) or ketamine with no additional interventions during infusion (control group; n=21). A comprehensive assessment protocol, including clinician-rated depression scales, depression-specific questionnaires, and open-ended qualitative descriptions of subjective experiences, was implemented at multiple timepoints. Through both qualitative and quantitative mixed methods analyses, this study sought to study the impact of this combined sensory intervention in the context of ketamine treatment for clinical depression, contributing clinically relevant insights to the evolving landscape of psychiatric care.

## 2 Materials and methods

### 2.1 Participants

Forty-three individuals with clinical depression were randomized to receive either ketamine plus mindfulness, music, and visual occlusion using eye masks (n=22) or ketamine only (n=21) (NCT05168735; a CONSORT diagram is provided in **Supplementary Figure S1**). Participants were adults (aged 19 to 65, 29 females, 13 males, one transgender male) who scored 14 or higher on the Hamilton Depression Rating Scale (HAM-D) at enrollment (28, 29). Additional key demographic and clinical characteristics of the study participants are provided in **Supplementary Table S1**. Patient recruitment for this study began on February 1, 2022, and the trial concluded upon successfully reaching the target sample size as planned within the allocated budget. Individuals with a lifetime history of bipolar disorder or psychosis or with current acute suicidality were excluded. Individuals with a substantial self-reported history of meditating with mindfulness techniques (>1 hour weekly on average for the past 6 months or longer) were also excluded in order to reduce the likelihood that those allocated to the control arm would spontaneously apply mindfulness meditation techniques. All enrolled participants met DSM-5 diagnostic criteria for major depressive disorder (n=40; 93%) or depressive disorder not otherwise specified (n=3; 7%); diagnoses were assessed via a MINI diagnostic interview administered at baseline by a trained interviewer, and final diagnostic determination was made by a licensed clinical psychologist (RBP). Participants were also excluded from the study sample if they reported any changes to their treatment regimen within four weeks of their baseline assessment. Full inclusion/exclusion criteria are available on the clinicaltrials.gov site (NCT05168735). The authors assert that all procedures contributing to this work comply with the ethical standards of the relevant national and institutional committees on human experimentation and with the Helsinki Declaration of 1975, as revised in 2013. All procedures involving human subjects/patients were approved by the University of Pittsburgh Institutional Review Board (STUDY21110040). All participants provided written informed consent.

### 2.2 Study design

All participants were randomized to undergo one of two pre-infusion training sessions (1): a 30-minute mindfulness training session that included a 20-minute guided mindfulness meditation and brief, scripted discussions with research staff regarding the objectives, obstacles, and potential advantages of mindfulness, both before and after the guided meditation; or (2) a 20-minute audio series of ‘academic exercises’ featuring diverse educational content such as excerpts from audiobooks detailing historical periods, cooking exercises, and prompts for silent engagement in mental arithmetic challenges (e.g., “serial 7s”). Participants in this second group also engaged in scripted conversations with research staff exploring the concept and potential benefits of ‘academics’ and ‘brain training’. This served as a control, accounting for both the cognitive demands and the social interactive elements present in the mindfulness arm. The training sessions familiarized the mindfulness group with their intervention while providing an equivalent pre-infusion timeline and level of attention and cognitive activation for the control group, allowing us to isolate the specific effects of mindfulness and ensure controlled comparisons. Both groups engaged in scripted discussions to ensure consistent social interaction, reduce confusion, and minimize confounding variables; both groups then completed cognitively engaging, active listening tasks that required concentration and mental focus, enabling a systematic exploration of the interventions’ specific effects when isolated from a range of non-specific effects. The mindfulness training script is provided in Appendix A.

After completing the assigned pre-infusion training session, participants immediately received a single 40-minute intravenous ketamine infusion (0.5mg/kg). During the infusion, participants in the combined sensory intervention group also listened to a predetermined playlist of classical music selections (informed by previous research with serotonergic psychedelics (SPs) (30) played through headphones; brief mindfulness reminder prompts, extracted from the same guided meditation used in pre-training, were interwoven with the music (see **Supplementary Table S2**). To ensure consistency in the experimental design, the same music and mindfulness tracks were used for all participants. Participants were not allowed to select their own music; given that the combination of mindfulness and music was being investigated, this could have influenced the results. Participants in this group also wore a light-occluding eye mask. The eye mask was intended to minimize external visual stimulation and support inward attentional focus, consistent with practices in mindfulness and psychedelic therapy research. In contrast, those in the control condition underwent the infusion with no specific instructions, music, other audio content, or additional enhancements, as in prior ketamine trials conducted by our group (e.g (31). In short, the control group received no interventions during the ketamine infusion, while the combined sensory intervention group received mindfulness and music during the ketamine infusion.

Participants in both groups responded to a range of questionnaires during the pre-infusion baseline and at different intervals post-infusion (at 24 hours and at 5, 12, 21, and 30 days post-infusion). The primary pre-specified clinical outcome for the trial was Montgomery-Åsberg Depression Rating Scale (MADRS) score (32). Additional details regarding the scales and secondary measures are provided in the Supplement.

It should be noted that missing data were limited in this study. Three participants did not have measurements at the 24-hour post-infusion time point, and one participant was missing data at days 21 and 30. No imputation was applied; analyses were conducted using the available data for each time point. Missingness was assumed to be missing at random (MAR), based on the study design and inspection of the data, which revealed no relationship between missingness and treatment group or baseline symptom severity.

### 2.3 Qualitative analysis

All participants were asked to describe their infusion experience in detail on day 30 post-infusion. A yes/no question assessed participant use of mindfulness techniques during the infusion. Nineteen of the 22 participants in the mindfulness group and 17 of the 21 in the control group provided responses, which were analyzed using Braun and Clarke’s six-stage framework (33). The qualitative analysis involved coding and identifying themes through a collaborative process among researchers. Formal test-retest reliability was not performed because the phenomena of interest were acute, time-sensitive experiences surrounding ketamine administration; thus, repeating interviews to assess stability risked altering, diluting, or retrospectively reconstructing the target experience. Member checking (participant validation of themes) was also not pursued to avoid unblinding and expectancy effects during the post-infusion follow-ups and to minimize participant burden.

Systematic coding procedures were used to enhance rigor and reliability despite these constraints, including a pre-defined thematic coding framework, iterative refinement, independent double-coding with adjudication, and calculation of intercoder agreement (Cohen’s κ = 0.78, indicating substantial agreement). Responses were stored securely and analyzed using Delve software (available at: https://delvetool.com/blog/codebook-qualitative-content-analysis). Additional details are provided in **Table 1** and in the Supplement.

**Table 1:** Qualitative outcomes of the study: main themes and subthemes, and quotes from two groups of participants with unipolar depressive disorders (n=36)* .

Themes	Subthemes	Definitions	Examples from transcripts of combined sensory intervention group	Percentage of participants in combined sensory intervention group reporting experience	Examples from transcripts of the control group	Percentage of participants in control group reporting experience
Diverse Sensory Experiences	Sensory alterations (auditory, visual, tactile)	Include audio, visual, and tactile alterations	• **Participant (P)1:** *‘In the beginning phases, the red numbers in the digital clock were white. I didn’t know what time it was until the very end because I would see numbers in doubles, triples, quadruples, or reverse … Sometimes the sound of the IV machine would make all kinds of noises or tones that made it seem like I could’ve been in some* sp*aceship.’* *•* ***P2:*** *‘My vision was affected and I found it unpleasant to focus my eyes – when I looked at anything directly, my field of vision would bounce or float in a sort of circular motion, constantly.’*	**12 (63%)**	• **P23:** *‘The ceiling was my focus as I could ‘see it shifting’. The center tiles I focused on stayed still and the others seemed to shift in a continuous pattern.’* • **P24:** *‘The colors behind my closed eyelids were nice.’*	**14 (82%)**
	Body distortion	Refers to a perceptual or cognitive discrepancy between one’s perceived body image and physical appearance.	• **P4:** *‘My arms and legs felt long and I felt like I was breathing very deeply and my chest was expanding bigger than usual.’* *•* ***P3:*** *‘I also felt like my body was shrinking and getting heavier.’*	**2 (10%)**	• **P26:** *‘I felt like I was floating at different points, suspended.’* • **P27:** *‘I began to feel very cosy and heavy in the bed and my limbs felt weighted in place.’*	**5 (29%)**
Engagement and Connection to Reality During the Experience	Engagement with the experience	When participants discuss engagement with the mindfulness instructions or using other techniques (either positive or negative) *ex. the instructions helped with breathing techniques*	• **P5:** *‘The mindfulness techniques worked to stabilize the experience and focus it on myself rather than the strangeness of the effects.’* *•* ***P7:*** *‘It was helpful. It helped me stay focused on the experience.’*	**10 (52%)**	• **P28:** *‘I didn’t use any mindfulness techniques, but just kept telling myself to pay attention as non-judgmentally as possible.’*	**1 (5%)**
	Connection to reality through mindfulness and music or other techniques	A connection to reality or sense of being present that occurs with the help of mindfulness instructions, music, or other techniques like breathing *ex. I was able to bring myself back to the present moment*	• **P8:** *‘It felt very easy to be more present with what was happening.’* *•* ***P3:*** *‘The music and audio recording were helpful. I was able to focus on the mindfulness techniques for the most part, and when my focus drifted I was able to be aware and bring it back to the mindfulness techniques.’*	**8 (42%)**	• **P29:** *‘I tried to be aware of what I was experiencing, while also aware of the surrounding in the room. I felt my breath was deeper, smoother and stronger.’*	**1 (5%)**
Experiencing Different Levels of Depression and Anxiety from Baseline	Break from depression or anxiety	A break from typical feelings of sadness or hopelessness, feeling a return to “normalcy”, and feeling more relaxed and peaceful	• **P9:** *‘My anxiety went away slowly.’* *•* ***P10:*** *‘I felt at peace with myself and the world for the first time in my life.’*	**15 (78%)**	• **P24:** *‘I felt at peace for the first time possibly ever.’* • **P30:** *‘I felt less connected to my body and my problems and was granted a sense of peace and tranquility unlike anything else.’*	**9 (52%)**
	Heightened anxiety and fear at some point of the experience	Stronger feelings of fear, and anxiety	• **P11:** *‘I felt my heart racing through some of it, especially when I started freaking out, wondering if I was going to have an anxiety attack during the experience … When I was able to get on top of my thoughts though, it was really beautiful … It was mostly going great even when my anxiety would kick in over the ‘high’ I felt.’* *•* ***P1:*** *‘Began to feel anxious at one point, because I was breathing so slowly. Then quickly, a wash of reassurance flowed over me that I would be fine.’*	**4 (21%)**	•	**0 (0%)**
Contradicting Experiences	Ability to tame negative thoughts (mindfulness)	Greater awareness of thoughts and the ability to release them	• **P1:** *‘There were many instances when just as things began to get too bizarre or scary, an immediate feeling of comfort and peace would follow.’* *•* ***P13:*** *‘When I tried to have negative thoughts, I saw them as silly and humorous rather than real.’*	**8 (42%)**	• **P31:** *‘Instead of my normal reaction which would be to panic and seek help from people nearby I kind of just laughed it off and didn’t make things worse.’* • **P32:** *‘I felt kind thoughts about myself and I was hopeful that things would get better.’*	**4 (23%)**
	Lack of control of thoughts (mindlessness, spiraling)	Feeling like you are unable to control your thoughts or that you are getting lost in them	• **P15:** *‘It was difficult at times to maintain the thoughts of the moment when it felt like things were expanding and or shrinking.’* *•* ***P2:*** *‘The main challenges to mindfulness during the infusion were getting lost in thought and also* sp*acing out and forgetting to focus on my breathing.’*	**5 (26%)**	• **P34:** *‘Hard to concentrate on any one thing.’* • **P23:** *‘I tried to control my focus and concentrate, but it seemed impossible to get myself to feel grounded.’*	**2 (11%)**
	Altered awareness of the physical body (decreased awareness vs. increased awareness)	Participants described heightened awareness or not feeling aware of physical sensations or the physical body	• **P6:** *‘The techniques discussed were useful about letting your thoughts go and just focusing on your body.’* *•* ***P1:*** *‘It didn’t feel like I had a body.’*	**6 (31%)**	• **P30:** *‘In some way, I felt less connected to my body.’* *•* ***P26:*** *‘I never felt like I completely left my body, but instead like I’d accessed a higher level of physical being, free of pain, hunger, worry.’*	**8 (47%)**
Awe and Spiritual Experiences	Altered perception of time (slow and fast)	The perception that time is speeding up or slowing down	• **P6:** *‘I had the ‘slowed-down time’ sensation that I associate with a marijuana high.’* *•* ***P2:*** *‘The duration of the infusion felt a lot shorter than it actually was, but it still felt like I went on a ‘journey’ of sorts.’*	**4 (21%)**	• **P29:** *‘I felt time went slower.’* • **P36:** *‘Time seemed to move quickly.’*	**7 (41%)**
	Self-diminishment (awe)	A feeling that one’s self or problems are small	• **P16:** *‘my physical being was encapsulated and unimportant.’* *•* ***P15:*** *‘The experience provided almost like a mental reset. It made me examine my problems in comparison to the world and universe itself and everything just began to seem small and insignificant again.’*	**5 (26%)**	• **P37:** *‘I felt very small in the universe.’* • **P25:** *‘I remember feeling very much a part of the universe.’*	**2 (11%)**
	Vastness (awe)	Feeling of something larger than one’s self or being in the presence of something grand	• **P8:** *‘It felt like a* sp*ace that held a lot of opportunity.’* *•* ***P13:*** *‘I felt like I was being let in on a great secret.’*	**5 (26%)**	• **P37:** *‘I was observing the world from another plane of existence.’* *•* ***P33:*** *‘I felt like I was in the presence of something divine.*	**3 (17%)**
	Connectedness (awe)	A feeling that one is connected to the other beings and/or the world at large or a feeling that things in the universe are connected	• **P9:** *‘I felt very calm and whole, without having a defined boundary between self and other.’* *•* ***P1:*** *‘I felt connection with other people and things.’*	**6 (31%)**	• **P25:** *‘I was connected to everything.’* • **P26:** *‘I experienced a deep connected-ness of all living things’*	**2 (11%)**
	Need for accommodation (awe)	Altering one’s existing schemas or ideas due to new information or new experiences *ex. experience that challenged existential thoughts*	• **P9:** *‘I felt that my paradigms of reality and creativity had changed; that there was so much more to these concepts than I had previously considered.’* *•* ***P17:*** *‘A sense of being, not of dread. Ability to smile, to handle tough situations a bit better.’*	**4 (21%)**	• **P27:** *‘I imagined I was receiving an upgrade like a new operating system.’* • **P33:** *‘At the end of the infusion I felt a refreshing and lingering sense of well being that I can still feel while recalling the experience.’*	**3 (17%)**
Challenges and Discomfort with the Experience	Discomfort with the experience	Participants shared negative feelings	• **P2:** *‘I remember that there was one piece of music that evoked feelings of mild annoyance.’* *•* ***P14:*** *‘I think the music playing was very distracting and kind of uncomfortable. I wouldn’t say I was relaxed, more so trying to decipher what was going on.’*	**4 (21%)**	• **P28:** *‘Irritating is the word that keeps coming to mind - the light, how stupid and derivative it was.’*	**1 (5%)**

*Nineteen of the 22 participants in the combined sensory intervention group and 17 of the 21 participants in the control group provided written responses and were included in the qualitative results.

P, Participant; Numbers indicate different participants.

### 2.4 Quantitative analysis

Two-tailed non-parametric tests of independent samples, specifically the Mann-Whitney U test, were used for each of these comparisons, as the variables did not follow a normal distribution (checked with the Kolmogorov–Smirnov test).

For the main depression rating scales—the MADRS and HAM-D—changes from pre-infusion baseline were also explored for both study groups. Pre-infusion baseline total scores were compared to each of the five post-infusion total scores (at 24 hours and at 5, 12, 21, and 30 days post-infusion) separately in the combined sensory intervention and control groups. Two-tailed non-parametric dependent tests of Wilcoxon signed ranks were used for this analysis. Results from parametric paired sample *t*-tests, as well as from a more generalized method (Generalized Estimation Equation (GEE)), are also reported in the Supplement for the same analyses (see **Supplementary Tables S4-1, S4-2**, **S5-1, S5-2**). GEE was used instead of a linear mixed model because it is more robust to various distributions, while linear mixed models are more suitable for normally distributed data (34). Moreover, the GEE approach is robust to unbalanced data and, under the MAR assumption, can yield unbiased parameter estimates without requiring imputation. This made it well-suited for the present study, where missingness was minimal and unrelated to treatment allocation or baseline symptom severity. In the GEE, the MADRS or HAM-D were used as the dependent variable, with participant groups and time intervals as factors. All results from these different approaches were consistent with each other. As an exploratory analysis, potential latent group differences in clinical outcomes were also examined using a Fisher’s exact test—specifically, rates of response, remission, and worsening. This approach was inspired by the responder/remitter analysis framework used by Carhart-Harris and colleagues (35).

To assess the internal consistency of the clinician-rated depression scales in our sample, Cronbach’s alpha was computed for the MADRS and HAM-D. Reliability was high for both scales (MADRS: α = 0.77; HAM-D: α = 0.80), consistent with prior reports of their psychometric robustness in clinically depressed populations. To examine stability across conditions, alphas were also computed by group and timepoint (see **Supplementary Table S3**). Values were comparable across the intervention versus control groups and baseline versus post-infusion assessments, indicating consistent reliability in this sample. In addition, a sensitivity power analysis was conducted using G*Power (36) in order to assess whether the study was adequately powered to detect meaningful effects. The analysis indicated that the current sample size (n=22 in the experimental group; n=21 in the control group) was sufficient to detect a minimum effect size of Cohen’s d=0.88 with 80% power at an alpha level of 0.05 (two-tailed), using a between-groups *t*-test. All analyses were conducted in MATLAB 2021 and SPSS 29.

## 3 Results

To promote a comprehensive understanding of the potential benefits and nuances associated with the integration of combined sensory intervention during ketamine infusion, the outcomes of this study are presented through two distinct lenses: qualitative and quantitative results.

### 3.1 Qualitative results

During ketamine infusions with or without the mindfulness, music, and eye mask intervention, participants described diverse experiences, including sensory changes (auditory, visual, and tactile), body distortion, and emotional fluctuations. Themes included connection to reality, breaks from depressive symptoms, heightened anxiety, and managing negative thoughts through mindfulness. Some reported loss of control, altered body awareness, time distortion, awe, and occasional discomfort. Further details and participant quotes are provided in **Table 1**.

Both groups experienced predominantly common themes, indicating consistent core effects of ketamine. However, there were some notable differences. For instance, only two participants (11%) in the control group reported using techniques to stay aware of the experience and remain connected to reality (‘Engagement and Connection to Reality’ theme), versus 52% of participants in the combined sensory intervention group who noted that these techniques helped them stabilize and focus on their experience rather than the strangeness of it. In addition, 42% of those in the combined sensory intervention group reported that these enhancements helped them stay present and aware of what was happening, underscoring that combined sensory intervention techniques might enhance participants’ ability to stay grounded during the infusion experience.

The ‘ Experiencing Different Levels of Depression and Anxiety From Baseline’ theme also differed between the two groups. Specifically, 78% of participants in the combined sensory intervention group reported a break from depressive symptoms and anxiety, though 21% also experienced short periods of fear and anxiety during the infusion, often attributable to reasons such as fear of slow breathing or the strangeness of the experience; no participants attributed this fear to the combined sensory intervention itself, and the feelings of fear and anxiety quickly subsided. In contrast, while fewer participants in the control group (54%) reported a break from depressive symptoms and anxiety, none reported challenging experiences of fear and anxiety during the infusion. This suggests that music and mindfulness instructions evoked varying responses, potentially leading to heightened emotional sensitivity and the experience of a wider emotional spectrum, including both higher and lower anxiety and fear. As noted above, however, the combination of mindfulness, music, and eye mask appeared to help participants in the combined sensory intervention group stay connected to reality, and this connection may have enabled them to reduce anxiety quickly by recognizing the experience as transient and non-threatening.

Furthermore, participants in the combined sensory intervention group reported a greater ability to tame negative thoughts compared to the control group (42% vs. 23%). This suggests that even though participants in the combined sensory intervention group experienced fear and anxiety at certain timepoints, they were able to manage these negative emotions and ultimately had a positive overall experience. This is consistent with the occurrence of adverse events (see **Supplementary Table S4**); only two of the 22 participants (9.09%) from the combined sensory intervention group and one of the 21 participants (4.76%) from the control group reported new or worsening anxiety from the pre-infusion baseline, indicating that there was no difference in the frequency of anxiety symptoms between the groups.

Notably, participants in both groups reported predominantly positive experiences, though the combined sensory intervention group reported a higher frequency of positive experiences. For instance, the following themes were endorsed more often by those in the combined sensory intervention group than the control group: ‘Engagement with the Experience’ (52% vs. 5%); ‘Connection to Reality’ (42% vs. 5%); ‘Break from Depression or Anxiety’ (78% vs. 52%); and ‘Ability to Tame Negative Thoughts’ (42% vs. 23%). Conversely, transient negative experiences were also more frequently reported by the combined sensory intervention group. Specifically, ‘Lack of Control of Thoughts’ and ‘Discomfort with the Experience’ were endorsed more often by those in the combined sensory intervention group than the control group (26% vs. 11% and 21% vs. 5%, respectively). As noted above, experiencing anxiety and fear at a particular moment during the experience was exclusively reported by participants in the combined sensory intervention group (21%), suggesting that these interventions heightened awareness and emotional sensitivity, making participants more attuned to both the positive and negative aspects of their experience. Overall, these findings suggest that a combined mindfulness, music, and eye mask intervention can intensify the overall experience, enhancing both positive and negative emotional responses. The increase in positive experiences indicates that these interventions might be beneficial in promoting engagement, connection to reality, and managing negative thoughts. However, the higher incidence of negative experiences, such as a lack of control over thoughts and experiencing anxiety and fear at particular moments, highlights the complexity of these interventions and the need for careful monitoring and support to manage participants’ intense emotional responses.

### 3.2 Quantitative results

**Figure 1:**
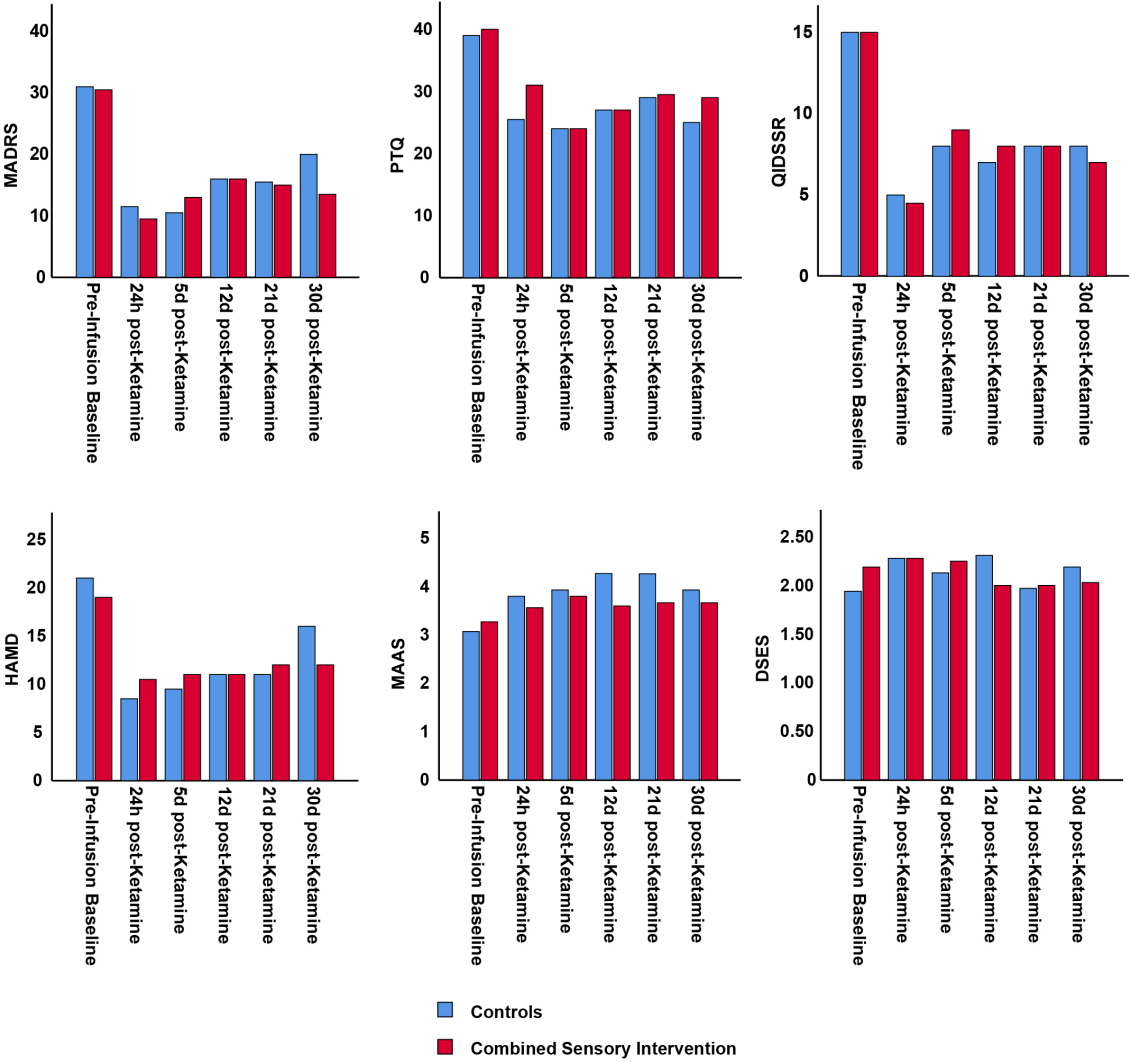
MADRS score (primary outcome measure) and secondary outcome measures (HAM-D, QIDS, MAAS, PTQ, and DSES) in participants who received the mindfulness, music, and eye mask intervention during ketamine infusion compared to controls who received only ketamine without any additional interventions. Scores for six different scales are illustrated for participants who received ketamine plus mindfulness, music, and eye mask (combined sensory intervention, red) or controls (blue) at pre-infusion baseline and at five different timepoints post-ketamine infusion. No significant differences were found between individuals in the combined sensory intervention group and the control group. MADRS, Montgomery-Asberg Depression Rating Scale; HAM-D, Hamilton Depression Rating Scale; QIDS, Quick Inventory of Depressive Symptomatology; MAAS, Mindful Attention Awareness Scale; DSES, Daily Spiritual Experience Scale; PTQ, Perseverative Thinking Questionnaire.

A comparison of the two groups on the primary outcome measure (**Figure 1**), the secondary outcome measures, and the other scales (**Supplementary Figures S3**–**S5**) identified no significant differences at any timepoint. Specifically, outcomes for individuals in the combined sensory intervention group did not differ from those who received ketamine alone (preceded by educational clips), at least in the context of depression rating scale scores and other measured psychological outcomes. When changes in MADRS and HAM-D scores were compared between pre-infusion baseline and each post-infusion timepoint, no significant differences were observed between the groups, underscoring that even variations in depression rating scale scores from pre-infusion baseline to post-infusion were similar between the two groups (**Supplementary Figure S2**).

**Figure 2:**
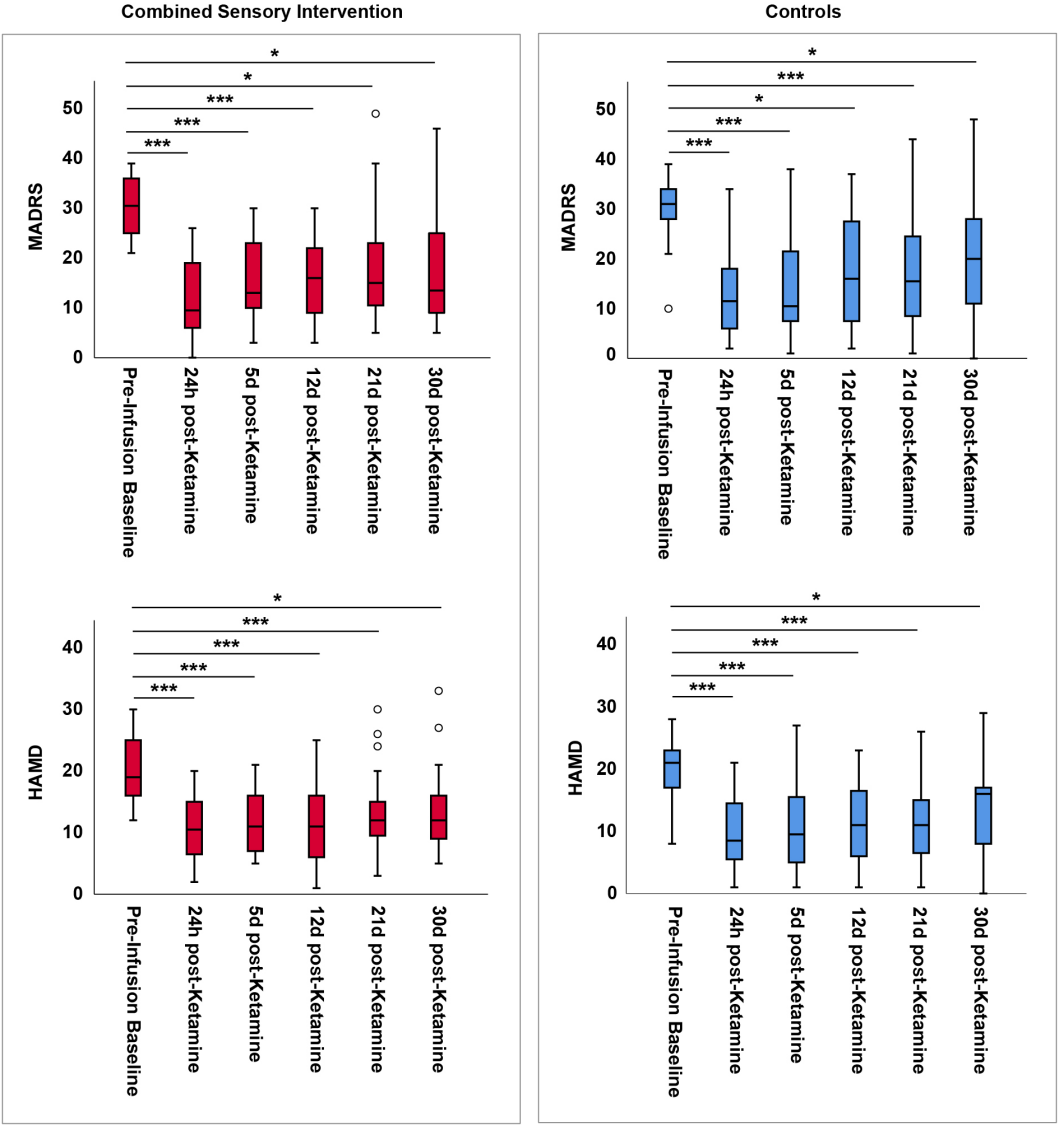
Boxplot representation of Montgomery-Asberg Depression Rating Scale (MADRS) (primary outcome measure) and Hamilton Depression Rating Scale (HAM-D) (secondary outcome measure) scores for participants who received ketamine plus mindfulness, music, and eye mask (combined sensory intervention, red, left plots) or controls who received only ketamine without any additional interventions (blue, right plots) across pre-infusion baseline and five post-ketamine timepoints. The boxplots demonstrate a significant decrease in both MADRS and HAM-D scores at each of the five post-infusion timepoints compared to the pre-infusion baseline in both groups. Statistical significances (after Bonferroni correction) are denoted by asterisks: ***p < 0.001; *p<0.05.

As expected, depressive symptoms as assessed by the MADRS and HAM-D total scores were significantly reduced at all five post-ketamine timepoints for both groups (mostly with p<0.001). Results from both non-parametric (Wilcoxon Signed Rank test) and parametric (paired samples *t*-test) analyses are presented in **Supplementary Tables S5-1**, **S6-1**, while **Supplementary Tables S5-2**, **S6-2** provide additional modeling using GEE to account for within-subject variability across time points (see also **Figure 2**). In this context, ketamine rapidly (as soon as 24 hours post-infusion) and robustly decreased depressive symptoms relative to pre-treatment baseline, regardless of whether it was administered in conjunction with the combined mindfulness, music, and eye mask intervention or not; these effects were sustained, to a lesser magnitude, throughout the remaining timepoints, including 30 days post-infusion.

No differences in anxiety scores, as assessed by the Visual Analogue Scale (VAS), were found when the combined sensory intervention and control groups were compared at 40, 80, or 120 minutes post-infusion, indicating that levels of self-reported anxiety were the same post-infusion. However, given that baseline anxiety levels could differ among participants, changes in anxiety at 40, 80, and 120 minutes post-infusion relative to baseline levels were also examined. Again, no significant differences were observed in these relative anxiety changes, indicating no difference between the two groups. Results of the exploratory analysis assessing latent group differences in clinical outcomes are presented in **Supplementary Table S7**. No significant differences were observed between the groups in terms of response (Fisher’s exact test: p=1.000; χ² (1)=0.114, p=0.736) or remission (Fisher’s exact test: p=0.752; χ² (1)=0.400, p=0.527) rates.

## 4 Discussion

Collectively, the results of this study underscore that integrating mindfulness, music, and visual occlusion (eye mask) into ketamine treatment can profoundly shape the patient experience and enrich the emotional and subjective dimensions of the infusion process. Qualitative analyses showed that participants who received the mindfulness, music, and eye mask intervention during the infusion often described a deeper engagement with the experience, marked by moments of relief, clarity, connection, and even awe—emotional undertones reported less frequently by the control group. Interestingly, quantitative analyses showed that depression scores improved significantly and similarly across both groups at different post-infusion timepoints (24 hours and 5, 12, 21, and 30 days post-infusion), suggesting that combined sensory intervention—including mindfulness, music, and a light-occluding eye mask—did not affect antidepressant response to ketamine as assessed by depression rating scale scores.

Quantitative analyses found no significant differences in depression scale scores between participants who received ketamine with the combined mindfulness, music, and eye mask intervention compared to a control group who received ketamine alone. This aligns with a study of 37 individuals with treatment-resistant depression who underwent 494 sessions of intranasal esketamine (37). In 52% of these sessions, patients listened to their own music via headphones. While the study did not directly contrast participants who listened to music with those who did not, no disparity was found in MADRS or Beck Depression Inventory (BDI) scores between the two groups; notably, however, those who listened to music had reduced anxiety, lower blood pressure, and better tolerance to higher doses. Our quantitative results are also in line with a recent study by Greenway and colleagues (23) who similarly found no significant differences between ketamine combined with music and a mindfulness-supportive control condition (no eye mask), compared to ketamine alone. In their study of individuals with severe, highly comorbid treatment-resistant depression, mindfulness was delivered as non-standardized encouragement to engage in practices such as body scanning and breath awareness. Together, these studies highlight the complexity of parsing the specific contributions of experiential adjuncts to ketamine therapy and point to the need for further trials with larger samples, more structured interventions, and standardized control conditions.

The study hypothesis was that the combined sensory intervention would yield greater reductions in depressive symptoms than ketamine alone, via mechanisms that leveraged ketamine’s neurobiological window of enhanced plasticity (via BDNF upregulation and mTOR pathway activation) and supported emotion regulation processes. Contrary to this expectation, quantitative measures (MADRS, HAM-D) did not differ significantly between groups, though qualitative reports indicated that participants receiving the intervention experienced more tolerable, emotionally grounded, and enriched infusions. This divergence suggests that such adjuncts may primarily influence the subjective quality and integration of the ketamine experience rather than producing large, immediate changes in symptom severity within the measured time frame. However, it should be noted that the modest sample size may have limited statistical power to detect smaller between-group effects in quantitative outcomes.

In this study, all participants (both groups) exhibited significantly reduced MADRS and HAM-D scores 24 hours post-ketamine infusion and at five other post-ketamine timepoints compared to pre-infusion baseline scores. Ketamine’s antidepressant effects have been well-documented in randomized, controlled trials (3). Because all participants and evaluators in this study were aware of the open-label ketamine infusion, the decreased depression scores likely reflect a combination of ketamine’s therapeutic effects and non-specific factors such as patient and rater expectancies, repeated assessment, and social attention from the study team, carefully matched across conditions.

Broadly, the qualitative findings identified a variety of both positive and negative effects for participants of both groups, though predominantly positive experiences were noted. Despite differences in the interventions, participants in both groups experienced similar broad themes (see **Table 1**). This suggests that while a combination of mindfulness, music, and eye mask may enhance certain aspects of the experience, neither was necessary for achieving a robust antidepressant response; furthermore, the core effects of the ketamine infusion were consistent across both groups, aligning with our quantitative findings showing no differences in depression rating scale scores between the groups. Some of these experiences—both positive and negative—have been noted in prior studies exploring the experiential nature of a ketamine infusion without additional interventions. For example, body distortion (38, 39) and sensory alterations (mostly visual) (40) have previously been reported with ketamine infusion and appear to be intrinsic to the drug itself, although they can also be affected by music and mindfulness (12). For instance, a handful of studies exploring the effects of music on ketamine infusion found that hearing music during ketamine treatment altered the treatment experience, with some finding comfort in specific music genres, while others felt that music kept them more connected to the external world (16, 22). Although the previous literature exploring music or mindfulness alongside ketamine is scant and encompasses diverse medical conditions (20), medication combinations, dosages, and order of administration (16), several studies have highlighted the beneficial impact of music in conjunction with ketamine for individuals with mood disorders. Specifically, such studies suggest that music or mindfulness administered in conjunction with ketamine reduced participant distress surrounding dissociative symptoms, providing a relaxing effect that facilitated subsequent treatments (6, 7, 18–21). However, in the current randomized trial, no noteworthy differences were observed across the two groups with regard to adverse events and acute mood states post-infusion. In addition, the use of a light-occluding eye mask in our intervention group may have contributed to participants’ inward focus and modulation of sensory input. Recent findings from Farnes and colleagues (27) demonstrated that visual occlusion during ketamine infusion altered EEG signal complexity and was associated with distinct phenomenological profiles—such as increased visual imagery and reduced anxiety—compared to eyes-open conditions. These effects may help explain participants’ reports of heightened emotional sensitivity in the present study and suggest that eye mask use could be a non-pharmacological means to shape the therapeutic experience.

As noted above, in the present study, both positive and negative experiences were more frequently reported by the combined sensory intervention group. Broadly, participants in the combined sensory intervention group appeared to experience heightened emotional sensitivity, making them more attuned to both positive and negative aspects of their experience. Some of these reactions may have been a direct result of mindfulness techniques; for instance, encouraging participants to note their thoughts or emotions in a non-judgmental way may have made them more likely to notice that they could not ‘control’ their thoughts. Similarly, such noting of the experiential state may have made participants more likely to recognize and label their discomfort, fear, or anxiety and recall it when prompted 30 days later. The inability of participants to select their own music may also have led to discomfort, as personalizing music is often recommended in ketamine therapy (6, 16, 19). An additional exploratory analysis was conducted to explore potential relationships between the qualitative and quantitative findings in this study. The analysis sought to assess whether there were differences in MADRS and HAM-D score changes between participants in the combined sensory intervention group who reported specific themes during the qualitative analysis compared to those who did not, as well as compared to the control group (see **Supplementary Figure S6**). Although the results from this analysis did not reach statistical significance, they may point to potential areas for further research, particularly in studies with larger sample sizes.

From a mechanistic perspective, the heightened emotional responses observed in some participants may reflect a synergistic engagement of neural and psychological systems. Ketamine rapidly reduces amygdala-hippocampal reactivity to negative stimuli and enhances prefrontal-limbic connectivity, creating a neuroplastic window whereby environmental inputs—such as music or mindfulness or eye mask—can exert especially potent effects on emotion processing (e.g., decreased amygdala reactivity to negative images correlates with increased connectivity to the pregenual anterior cingulate cortex) (41). Mindfulness training engages interoceptive and emotion-regulation regions, including the insula, anterior cingulate cortex, and prefrontal cortex, enhancing awareness and labeling of emotional states (42). Concurrently, music stimulates affective subcortical circuits such as the amygdala and hippocampus, anchoring emotional experiences in a sensory context. These interventions may amplify emotional salience during ketamine’s neuroplastic phase, leading to more intense subjective experiences in some individuals. However, inter-individual differences (such as baseline neural connectivity, emotion regulation capacity, or prior mindfulness familiarity) could explain why some participants had stronger emotional reactions than others. For instance, those with greater baseline amygdala-prefrontal coupling might be more sensitive to emotion-laden stimuli post-infusion, making them particularly responsive to music or mindfulness cues. To disentangle these mechanisms, future studies should incorporate neuroimaging (e.g., fMRI, EEG) to elucidate how these interventions modulate brain networks in real time, thus clarifying both common and individual-specific pathways. Such data would strengthen the theoretical contribution, linking changes in neural dynamics to qualitative reports of positive or negative emotional responses.

The present findings serve to highlight a key point regarding the role that therapeutic milieu may play in the psychoactive differences between ketamine and SPs such as psilocybin, lysergic acid diethylamide (LSD), or 3,4-Methylenedioxymethamphetamine (MDMA). This observation underscores the critical need for a multi-disciplinary approach in psychedelic-assisted therapies, where the expertise of psychologists and mental health professionals may influence therapeutic outcomes (7, 16, 19). The concepts of set (mindset) and setting (that is, where the agents are administered) have been implicated in therapeutic outcomes following SPs (43), although direct causal evidence for their impact on therapeutic outcomes remains limited. While correlational data support the relationship between acute subjective experiences and clinical response—for both SPs and ketamine—randomized trials that experimentally manipulate these contextual factors are still underway, and data are not yet available (44, 45). Ketamine is typically administered in standard clinical settings with relatively less focus on set and setting than SPs, which are often delivered in more controlled, supportive environments (46–49). In this context, our study adds to the growing body of research by experimentally manipulating components of the ketamine therapeutic environment—specifically, mindfulness, music, and eye mask—and observing their effects on the subjective experience. Here, mindfulness, music, and eye mask added new dimensions to the ketamine experience, as those who received the combined sensory intervention during ketamine more often reported positive effects, such as connecting to reality and managing negative thoughts, compared to those who received ketamine alone; this suggests that similar interventions may potentially enhance patient adherence to future treatments.

It is important to note that the mindfulness intervention used in this study was limited to a single 20-minute audio-guided session, which likely represents a minimal dose relative to protocols shown to produce robust clinical effects. Brief mindfulness inductions, particularly among mindfulness-naïve individuals, have been shown to yield only modest and often conditional state-level effects (50). This limited exposure may have attenuated potential differences between the intervention and control groups. Future studies may benefit from incorporating extended or repeated mindfulness training protocols, such as those used in Mindfulness-Based Cognitive Therapy (MBCT) (51), to better assess synergistic effects with ketamine. Early evidence drawn from other clinical populations, such as substance use disorders, suggests that more intensive mindfulness interventions delivered in conjunction with ketamine may enhance therapeutic outcomes (20).

Additional limitations should also be noted. First, the sample size was small (n = 43 randomized; 22 in the combined sensory intervention group versus 21 in the controls). A sensitivity power analysis indicated that this sample provided 80% power (α = 0.05, two-tailed) to detect a minimum effect size of Cohen’s d = 0.88. While this suggests adequate power for large effects, smaller but potentially meaningful differences may have gone undetected. Larger, adequately powered trials are needed to confirm and extend these findings. Second, the inclusion of a third group receiving drug placebo would have provided a more comprehensive understanding of ketamine’s effects. Third, individual variability in baseline depression severity, prior exposure to ketamine, and familiarity with mindfulness practices were not systematically assessed or controlled for in the present analyses. These factors may have influenced both subjective experiences and treatment response and should be considered in future work. Fourth, adding brain imaging techniques such as magnetoencephalography or electroencephalography could have helped further elucidate the effects of our combined therapeutic approach. Physiological measures (e.g., heart rate variability, skin conductance) could also offer additional insight into emotional arousal and regulatory processes during and after ketamine infusion. Fifth, the use of open-ended questionnaires, which was useful for gathering data from nearly all participants quickly, may nevertheless have led to variability and missed nuances in responses. Future studies might benefit from semi-structured interviews to better capture the details of the ketamine infusion experience. Sixth, to maintain consistency in our experimental design, the same music and mindfulness tracks were played for all participants. While this standardization facilitated control across participants, it may have reduced the intervention’s effectiveness for some participants, particularly those unfamiliar with mindfulness or who found the provided music less engaging or even discomforting. This variability in personal resonance with the content may have contributed to the mixed qualitative reports; prior work suggests that music tailored to individual preferences may be more effective in shaping ketamine experiences (6). Future research could explore personalized music selection and mindfulness scripts stratified by prior mindfulness experience or individual preference to better match participants’ backgrounds and optimize engagement. Seventh, test-retest interviews were not conducted. Given the acutely evolving ketamine experience and the need to minimize reactivity and burden, these procedures were not feasible within the study design. Future work should incorporate pragmatic approaches to participant validation (e.g., brief theme summaries for confirmation) and, where appropriate, short-interval retest interviews to assess thematic stability. Eighth, data on participants’ engagement in concomitant psychotherapy with external providers during the study period was also not collected. Prior naturalistic research suggests that concurrent psychotherapy may moderate the therapeutic effects of ketamine infusions on symptom reduction, with greater improvements observed among those receiving psychotherapy (52). Future studies should assess and account for such factors to better clarify their potential moderating influence. Finally, the time delay between infusion and participants sharing their experiences was 30 days, which allowed participants to reflect on the most salient aspects of the experience from a more distal, and potentially more crystallized or enduring, vantage point, but it may also have impacted their recollection of events.

In light of the promising subjective outcomes observed, these findings also carry potential implications for clinical practice. Clinicians considering the integration of mindfulness, music, and eye mask into ketamine treatment protocols may benefit from using brief, structured interventions timed to coincide with ketamine’s acute psychoactive window. For example, a 10-15 minute guided mindfulness session initiated shortly before infusion, followed by continuous ambient music throughout the infusion, may offer a feasible and low-burden approach that supports patient comfort and emotional processing, though these may not necessarily enhance quantitative antidepressant outcomes. Implementation in real-world settings may require minimal clinician training in delivering brief mindfulness scripts and access to curated, licensed music libraries; these steps are relatively low-cost and could be incorporated into existing ketamine protocols without substantial workflow disruption. Future research focused on real-world implementation should explore flexible protocols that allow for personalization, such as letting individuals select from a curated set of music genres or mindfulness styles (e.g., body scan vs. breath awareness) based on their preferences or therapeutic goals, while also allowing them to opt out without impacting the core ketamine treatment, as overall antidepressant efficacy appears unlikely to be impacted. Given the absence of significant quantitative effects, combined with the qualitative reports of reduced anxiety and enhanced emotional grounding, such adjuncts should be framed as optional enhancements aimed at improving patient experience rather than as established boosters of antidepressant efficacy.

In conclusion, this first randomized trial to test the impact of experimentally manipulating a combined sensory intervention—including mindfulness, music, and a light-occluding eye mask—during ketamine infusion found that depression scores improved significantly and similarly across both groups—regardless of whether participants received ketamine alone or in combination with mindfulness, music, and eye mask—highlighting ketamine’s robust therapeutic efficacy. However, qualitative data revealed that the combined sensory intervention meaningfully enriched the subjective experience for most—but not all—participants, heightening their awareness of positive emotional states such as deeper engagement, a stronger sense of connection to reality, greater focus on the present moment rather than its strangeness, and even episodes of relief, awe, and spiritual insight. A small number of participants also reported discomfort—an effect that might be mitigated by offering these interventions as an optional component.

Given the limited and largely anecdotal research into ketamine therapy combined with either mindfulness or music or eye mask, these findings are a valuable contribution to the field, laying the groundwork for future investigations in this area aimed at optimizing therapeutic context, integration, and adherence.

## Data Availability

The raw data supporting the conclusions of this article will be made available by the authors, without undue reservation.
