# Supplementary material for: Mindfulness, music, visual occlusion in ketamine therapy for depression: do they change outcomes? A qualitative and quantitative analysis of a randomized controlled trial
Source: Front Psychiatry. 2025 Sep 2;16:1642025. doi: 10.3389/fpsyt.2025.1642025 (PMC12439531; doi:10.3389/fpsyt.2025.1642025)
Supplement: Supplementary file 1 [file Supplementaryfile1.docx]

*Kheirkhah et al – Mindfulness, Music, and* *Visual Occlusion in Ketamine Therapy for Depression: Do They Change Outcomes? A Qualitative and Quantitative Analysis of a Randomized Controlled Trial*

**Supplementary Materials**

*Study Design*

One of a team of two experienced, Master’s-level, blinded outcome evaluators completed all evaluations at the pre-infusion and post-infusion timepoints for all participants—at the screening visit, at a pre-infusion baseline measured on the infusion morning, and at 24 hours, 5 days, 12 days, 21 days, and 30 days post-infusion. Most of these assessments were conducted remotely via videoconference, with the exception of the pre-infusion baseline and the 24-hour post-infusion assessments.

The random allocation sequence was generated in SAS (SAS Institute Inc., Cary, NC, USA), which randomly sorted the full list of possible allocations. Randomization was stratified by biological sex at birth, using permuted blocks of size n=2 or n=4. A single researcher, uninvolved in other aspects of the trial, uploaded the sequence into RedCap (Research Electronic Data Capture). On the morning of the infusion, the participant’s stratum (sex) was entered into RedCap, which retrieved the next sequential randomized allocation. A single unblinded research team member then implemented the assigned procedure, while all other team members remained unaware of the allocation until the trial concluded.

To maintain blinding, outcome assessors were not present during the infusion or intervention procedures. Participants were also blinded, as they received minimal information about the study’s hypotheses and were provided with a uniform cover story (e.g., “brain training”) for both the combined sensory intervention and control procedures. While participants experienced their assigned procedures firsthand, they remained unaware of whether they were in an "active" or "control" condition as defined by the study team. The State Mindfulness Scale (SMS), a self-report measure evaluating mindfulness of both body and mind (1), was administered 80 minutes after the ketamine infusion as the primary mechanistic measure, designed to capture any impact of the experimental manipulation on state mindfulness during the infusion experience. Ongoing interrater reliability checks were conducted at six-month intervals to ensure consistent ‘excellent’ reliability of both individual item scores and total scores (ICCs≥.9).

Secondary measures, collected at identical timepoints to the Montgomery-Asberg Depression Rating Scale (MADRS), included: the modified 17-item Hamilton Depression Rating Scale (HAM-D) (2, 3); the Quick Inventory of Depressive Symptomatology (QIDS), which assesses depressive symptom severity (4); the Mindful Attention Awareness Scale (MAAS), which measures the frequency of open and receptive attention to ongoing events and experiences (5, 6); the Daily Spiritual Experience Scale (DSES), which assesses ordinary spiritual experiences excluding dramatic mystical encounters (7); and the Perseverative Thinking Questionnaire (PTQ), which captures individuals’ reflections on negative experiences (8). Additional scales were included at select time points. These included the Hood Mysticism Scale (HMS), which measures subjective spirituality (9) and was collected at 40 minutes and 24 hours post-ketamine infusion, and the Awe Experience Scale (AWE-S), which measures the state of awe (10) and was collected at 80 minutes post-infusion. To provide comprehensive information on the impact of the contextual (mindfulness, music, and eye mask) manipulation, all scale scores were included in the quantitative analyses for this study. Adverse events were tracked using the Patient Rated Inventory of Side Effects (11) and the Clinician-Administered Dissociative States Scale (CADSS (12)). These events were tallied for all symptoms (including anxiety) reported as new or worsening from the pre-infusion baseline and with onset occurring during the infusion or within the subsequent 24-hour period (see Table S3). Additionally, Visual Analogue Scale (VAS) slider scales (0-100) were used to collect data at pre-infusion, 40 minutes, 80 minutes, and 120 minutes post-infusion for a range of emotions, including anxiety.

*Quantitative Analysis*

This study compared outcomes from various scales (HAM-D, MADRS, QIDS, MAAS, PTQ, and DSES) between the two participant groups at the pre-infusion baseline and five different post-infusion time points: 24 hours, 5 days, 12 days, 21 days, and 30 days. Three other scales were used at different time points; the HMS was administered at 40 minutes and at 24 hours post-ketamine, and the SMS and AWE scales were both administered at 80 minutes post-ketamine.

*Qualitative Analysis Details*

The following open-ended question was presented to all patients in both study groups on Day 30 post-infusion: "Please describe your experience during and shortly after the infusion in about 2 full paragraphs in the box below. While you are writing, please focus as much as possible on the experience itself, rather than what led up to it, what happened afterwards, or your interpretation of the experience. Try to be as descriptive and specific as possible." This prompt provided the open-ended text included in our qualitative analysis (see Table 1).

Participants were sent this question as part of a remote battery of questionnaires. Nineteen of the 22 participants (86%) who received the mindfulness, music, and eye mask intervention and 17 of the 21 participants (81%) in the control group provided written responses to the uniform prompt and were thus included in the qualitative analysis. Written statements were stored on a secured server and subsequently de-identified and entered into Delve, a software package designed to aid in qualitative research. Following the six-stage analysis framework established by Braun and Clarke (13), two researchers initially reviewed the transcripts to become familiar with the data. Coding and creation of a preliminary code-book were conducted via meticulous line-by-line examination of the transcripts. Three researchers independently coded the transcripts, after which coding discrepancies were discussed and resolved to reach a consensus. The codes were further analyzed to identify overarching themes, forming the basis for developing a thematic model. This process of theme identification was collaborative and iterative, involving the entire team of qualitative coders.

*Exploratory Analysis of Clinical Outcomes between Groups*

To explore the possibility of latent group differences in clinical outcomes, exploratory analyses were conducted that compared response, remission, and worsening rates between the two groups. Response was defined as a ≥50% reduction from baseline in either MADRS or HAM-D scores, and remission was defined as achieving a MADRS score ≤10 and/or a HAM-D score ≤7. Worsening was defined as a ≥25% increase in MADRS or HAM-D scores from baseline. These binary outcomes were analyzed using Fisher’s exact test, which is appropriate for small sample sizes. The results, shown in Table S6, demonstrated no statistically significant differences between the combined sensory intervention group and the control group across any of the three outcome categories. Response rates were 65% in the combined sensory intervention group and 70% in the control group (Fisher’s exact test: p = 1.000; χ²(1) = 0.114, p = 0.736), and remission rates were 55% vs. 45%, respectively (Fisher’s exact test: p = 0.752; χ²(1) = 0.400, p = 0.527). Worsening was not observed in either group. Collectively, the findings suggest that although mean outcome scores did not differ significantly between groups, clinical response and remission outcomes also showed no categorical separation.

**Supplementary References**

1. Tanay G, Bernstein A. State Mindfulness Scale (SMS): development and initial validation. Psychol Assess. 2013;25(4):1286-1299.

2. Hamilton M. A rating scale for depression. J Neurol Neurosurg Psychiatry. 1960;23(1):56-62.

3. Reimherr FW, Amsterdam JD, Quitkin FM, Rosenbaum JF, Fava M, Zajecka J, Beasley J, Charles M, Michelson D, Roback P, Sundell K. Optimal length of continuation therapy in depression: a prospective assessment during long-term fluoxetine treatment. Am J Psychiatry. 1998;155(9):1247-1253.

4. Rush AJ, Trivedi MH, Ibrahim HM, Carmody TJ, Arnow B, Klein DN, Markowitz JC, Ninan PT, Kornstein S, Manber R. The 16-Item Quick Inventory of Depressive Symptomatology (QIDS), clinician rating (QIDS-C), and self-report (QIDS-SR): a psychometric evaluation in patients with chronic major depression. Biol Psychiatry. 2003;54(5):573-583.

5. Brown KW, Ryan RM. The benefits of being present: mindfulness and its role in psychological well-being. J Pers Soc Psychology. 2003;84(4):822-848.

6. Carlson LE, Brown KW. Validation of the Mindful Attention Awareness Scale in a cancer population. J Psychosom Res. 2005;58(1):29-33.

7. Underwood L. Ordinary spiritual experience: Qualitative research, interpretive guidelines, and population distribution for the Daily Spiritual Experience Scale. Arch Psychol Relig. 2006;28(1):181-218.

8. Ehring T, Zetsche U, Weidacker K, Wahl K, Schönfeld S, Ehlers A. The Perseverative Thinking Questionnaire (PTQ): Validation of a content-independent measure of repetitive negative thinking. J Behav Ther Exp Psychiatry. 2011;42(2):225-232.

9. Hood Jr RW. The construction and preliminary validation of a measure of reported mystical experience. J Sci Stud Relig. 1975;14:29-41.

10. Yaden DB, Kaufman SB, Hyde E, Chirico A, Gaggioli A, Zhang JW, Keltner D. The development of the Awe Experience Scale (AWE-S): A multifactorial measure for a complex emotion. J Posit Psychol. 2019;14(4):474-488.

11. Rush AJ, Fava M, Wisniewski SR, Lavori PW, Trivedi MH, Sackeim HA, Thase ME, Nierenberg AA, Quitkin FM, Kashner TM, Kupfer DJ, Rosenbaum JF, Alpert J, Stewart JW, McGrath PJ, Biggs MM, Shores-Wilson K, Lebowitz BD, Ritz L, Niederehe G, STAR*D Investigators Group. Sequenced treatment alternatives to relieve depression (STAR*D): rationale and design. Control Clin Trials. 2004;25:119-142.

12. Bremner JD, Krystal JH, Putnam FW, Southwick SM, Marmar C, Charney DS, Mazure CM. Measurement of dissociative states with the clinician-administered dissociative states scale (CADSS). J Trauma Stress. 1998;11:125-136.

13. Braun V, Clarke V. Conceptual and design thinking for thematic analysis. Qual Psychol. 2022;9(1):3.

**Supplementary Table S1. Key demographic and clinical features of the study participants.**

| **Participant** | **Age** | **Race/ Ethnicity** | **Screening visit MADRS score** | **Screening visit HAMD score** | **Ran-domiz-ation** | **Current medication** | **Psych med list** | **Psych med num** |
| --- | --- | --- | --- | --- | --- | --- | --- | --- |
| P1 | 46 | White; Non-Hispanic/Non-Latino | 29 | 17 | M/M | Yes | SAM-e (over the counter) (1600mg/day) | 1 |
| P2 | 31 | White; Non-Hispanic/Non-Latino | 24 | 18 | M/M | Yes | Prozac (40mg/day). Modafinil (100mg/day with option to take extra 100mg PRN as wakefulness agent) | 2 |
| P3 | 25 | White; Non-Hispanic/Non-Latino | 31 | 21 | M/M | Yes | None | 0 |
| P4 | 50 | White; Non-Hispanic/Non-Latino | 33 | 23 | M/M | Yes | Effexor (150mg/day). Wellbutrin (250mg/day) | 2 |
| P5 | 21 | Mixed (Multi-racial); Hispanic (South American) | 17 | 14 | M/M | Yes | Propanolol (10mg PRN for anxiety) | 1 |
| P6 | 35 | White; Non-Hispanic/Non-Latino | 12 | 14 | M/M | Yes | Vyvanse (70mg/day) Wellbutrin (150mg/day) Lexapro (10mg/day) | 3 |
| P7 | 45 | White; Non-Hispanic/Non-Latino | 21 | 20 | M/M | Yes | Trintellix (10mg/day). Wellbutrin (400 mg/day). Vyvanse (10mg/day) | 3 |
| P8 | 31 | White; Non-Hispanic/Non-Latino | 25 | 19 | M/M | Yes | Adderall 5 mg every other day | 1 |
| P9 | 54 | White; Other Hispanic/Latino | 47 | 30 | M/M | Yes | Escitalopram Oxalate (20 mg/day). Adderall (10 mg/day) | 2 |
| P10 | 53 | White; Non-Hispanic/Non-Latino | 26 | 24 | M/M | Yes | Prozac (80mg/day) Wellbutrin (150mg/day) | 2 |
| P11 | 47 | Mixed (Multi-racial); South American | 20 | 16 | M/M | Yes | Amitriptyline (25mg/day) | 1 |
| P12 | 46 | White | 36 | 30 | M/M | Yes | Prozac (40mg/day). Klonopin (0.5mg PRN) | 3 |
| P13 | 30 | White; Non-Hispanic/Non-Latino | 33 | 17 | M/M | Yes | Sertraline (100mg/daily). Naltrexone PRN. Trazodone (50mg/day) Bupropion (300mg/day) | 4 |
| P14 | 26 | Black or African American; Non-Hispanic/Non-Latino | 21 | 16 | M/M | Yes | Nortriptyline (150mg/day) | 1 |
| P15 | 27 | White; Non-Hispanic/Non-Latino | 39 | 27 | M/M | Yes | Amphetamine salts XR (20mg/day). Aripiprazole (5mg/day). Sertraline (100mg/day) Bupropion HCL XL (300mg/day). Gabapentin (300mg/day) | 5 |
| P16 | 65 | White; Non-Hispanic/Non-Latino | 30 | 21 | M/M | Yes | Lexapro (5mg/day). Bupropion (300mg/day) | 2 |
| P17 | 49 | White; Non-Hispanic/Non-Latino | 39 | 30 | M/M | Yes | Cymbalta (120mg/day). Ativan (1mg PRN). Lamictal (375mg/day). Ambien XR (12.5mg/day) | 4 |
| P18 | 19 | Black or African American; Non-Hispanic/Non-Latino | 38 | 22 | M/M | Yes | Adderall (10mg up to 3x/day). Latuda (60mg/day) | 2 |
| P19 | 62 | White; Non-Hispanic/Non-Latino | 36 | 15 | M/M | Yes | Effexor (150mg/day) | 1 |
| P20 | 58 | White; Non-Hispanic/Non-Latino | 34 | 14 | M/M | Yes | Wellbutrin (450mg/day) | 1 |
| P21 | 28 | White; Non-Hispanic/Non-Latino | 36 | 29 | M/M | Yes | Propranolol (20mg/day). Risperidone (0.25 mg/day). Sertraline (250mg/day). Wellbutrin  (250 mg/day) | 5 |
| P22 | 32 | Mixed (Multi-racial); Non-Hispanic/Non-Latino | 30 | 18 | M/M | No | None | 0 |
| P23 | 21 | Mixed (Multi-racial); Other Hispanic/Latino | 23 | 23 | Control | Yes | Paxil (50mg/day). Adderall XR (30mg/day) | 3 |
| P24 | 62 | White; Non-Hispanic/Non-Latino | 30 | 22 | Control | Yes | None | 1 |
| P25 | 33 | White; Non-Hispanic/Non-Latino | 39 | 30 | Control | Yes | Adderall XR and IR (60mg/day). Klonopin (0.5mg PRN) | 2 |
| P26 | 33 | White; Non-Hispanic/Non-Latino | 37 | 26 | Control | Yes | Fluoxetine (60mg/day) | 1 |
| P27 | 59 | White; Non-Hispanic/Non-Latino | 31 | 22 | Control | Yes | Parnate (50mg/day). Ativan (1.5mg/day) Trazodone (100mg/day) | 3 |
| P28 | 32 | White; Non-Hispanic/Non-Latino | 27 | 21 | Control | No | None | 0 |
| P29 | 25 | Asian; Non-Hispanic/Non-Latino | 24 | 21 | Control | Yes | Zoloft (100mg/day) Buspirol (5mg/day) | 2 |
| P30 | 34 | White; Non-Hispanic/Non-Latino | 42 | 30 | Control | Yes | Effexor XR (150mg/day). Clonazepam (0.5mg PRN). Dextro-amphetamine XR (15mg/day). Dextro-amphetamine IR (10mg PRN) | 3 |
| P31 | 45 | White; Non-Hispanic/Non-Latino | 28 | 15 | Control | Yes | Venlafaxine (150mg/day). Abilify (2mg/day). Ritalin (5mg PRN) | 3 |
| P32 | 30 | Mixed (Multi-racial); Non-Hispanic/Non-Latino | 33 | 24 | Control | Yes | Adderall XR (20mg/day). Lamictal (150mg/day). Effexor (150mg/day). Wellbutrin (150mg/day). Trazodone (50mg PRN) | 6 |
| P33 | 47 | White; Non-Hispanic/Non-Latino | 25 | 21 | Control | Yes | Wellbutrin (450mg/day). Xanax (unknown dose PRN). Trazodone (unknown dose PRN). Cymbalta (unknown dose QD) | 4 |
| P34 | 36 | White; Non-Hispanic/Non-Latino | 25 | 22 | Control | Yes | Wellbutrin XL (150mg/day). Vitamin D supplement (for depression, QD). Melatonin (0.5 mg PRN) | 3 |
| P35 | 60 | White; Non-Hispanic/Non-Latino | 21 | 14 | Control | Yes | None | 0 |
| P36 | 22 | White; Non-Hispanic/Non-Latino | 29 | 19 | Control | Yes | None | 0 |
| P37 | 24 | White; Non-Hispanic/Non-Latino | 28 | 17 | Control | No | None | 0 |
| P38 | 32 | White; Non-Hispanic/Non-Latino | 31 | 23 | Control | Yes | Pristiq (50 mg/day). | 3 |
| P39 | 55 | White; Non-Hispanic/Non-Latino | 21 | 21 | Control | Yes | Effexor (150 mg/day) | 1 |
| P40 | 25 | White; Non-Hispanic/Non-Latino | 37 | 24 | Control | Yes | Zoloft (50mg/day) | 1 |
| P41 | 24 | White; Non-Hispanic/Non-Latino | 39 | 20 | Control | Yes | Vyvanse (30mg/day). Fluoxetine (60 mg/day). | 2 |
| P42 | 30 | White; Non-Hispanic/Non-Latino | 20 | 16 | Control | Yes | Trintellix (20mg/day)  Ritalin (27mg/day) | 2 |
| P43 | 28 | Black or African American; Non-Hispanic/Non-Latino | 29 | 20 | Control | Yes | Lexapro (15mg/day) | 2 |

Abbreviations: ER: extended release; HAM-D: Hamilton Depression Rating Scale; IR: instant release; MADRS: Montgomery-Asberg Depression Rating Scale; M/M: mindfulness, music, and eye mask intervention; PRN: as needed; QD: once daily dosing; XL: extended release; XR: extended release

**Supplementary Table S2:** Audio reference details for the combined sensory intervention (mindfulness, music, and a light-occluding eye mask) group.

| *Beginning* | *End* | *Song Title* |
| --- | --- | --- |
| **0 Minutes** | **3 Minutes 25 Seconds** | Antonio Vivaldi, Andante, Concerto RV 532 in G Major for 2 guitars, strings & continuo, Guitar Concertos, Los Romeros, Academy of St. Martin-in-the-Fields, Philips 412-624-2. |
| **3 Minutes 25 Seconds** | **7 Minutes 16 Seconds** | Antonio Vivaldi, Largo, Concerto RV93 in D Major for guitar, strings & continuo, Ibid |
| **7 Minutes 16 Seconds** | **9 Minutes 51 Seconds** | Antonio Vivaldi, Largo, Concerto RV 356 in A Minor for guitar, Ibid. |
| **9 Minutes 51 Seconds** | **13 Minutes 10 Seconds** | Paul Horn "Mumtaz Mahal", Inside the Taj Mahal, Kuckuck, LC2099 |
| **13 Minutes 10 Seconds** | **18 Minutes 44 Seconds** | Paul Horn "Shah Jahan", Inside the Taj Mahal, Ibid. |
| **18 Minutes 44 Seconds** | **20 Minutes 52 Seconds** | Ron Korb, "Flute Traveller (Alto Flute), Oasis Productions Limited, SOCAN NHCD 205. |
| **20 Minutes 52 Seconds** | **27 Minutes 31 Seconds** | JS Bach: Suite No. 3 (Bach) Brazilian Guitar Quartet, Delos B00004YR6P |
| **27 Minutes 31 Seconds** | **33 Minutes 15 Seconds** | Edward Elgar, "Nimrod", Enigma Variation #9, Leonard Bernstein, The Artist's Album, DGG 457 691-2. |
| **33 Minutes 15 Seconds** | **39 Minutes 19 Seconds** | Morten Lauridsen "O Magnum Mysterium", A Robert Shaw Christmas: Angels on High, Telarc20 CD-80461. |
| **39 Minutes 19 Seconds** | **44 Minutes 47 Seconds** | "Alleluia, Behold the Bridegroom", Anonymous (c. 18^th^ Cent.), Sacred Treasures III: Choral Masterworks from Russia and Beyond, St. Petersburg Chamber Choir, Hearts of Space, 025041111423. |
| **Various Clips (overlaid over music at the beginning, middle, and end of the audio file)** | **Four 35-70 second “reminder” instruction clips extracted from guided meditation mp3** | Diana Winston. “Complete Meditation Instructions” (2021, January 18). *UCLA Mindful Awareness Research Center*. **UCLAhealth.org/marc/mindful-meditations** |

**Supplementary Table S3:** Cronbach’s alpha values for the Montgomery-Åsberg Depression Rating Scale (MADRS) and the Hamilton Depression Rating Scale (HAM-D) across groups and timepoints. Reliability was calculated separately for the combined sensory intervention and control groups at six assessment points (pre-infusion baseline, 24 hours, 5 days, 12 days, 21 days, and 30 days post-infusion).

| Scale | Group | Timepoint | Cronbach’s α |
| --- | --- | --- | --- |
| MADRS | Combined sensory intervention | Pre-infusion Baseline | 0.721 |
| MADRS | Combined sensory intervention | 24 hours | 0.756 |
| MADRS | Combined sensory intervention | 5 days | 0.753 |
| MADRS | Combined sensory intervention | 12 days | 0.763 |
| MADRS | Combined sensory intervention | 21 days | 0.773 |
| MADRS | Combined sensory intervention | 30 days | 0.778 |
| HAM-D | Combined sensory intervention | Pre-infusion Baseline | 0.810 |
| HAM-D | Combined sensory intervention | 24 hours | 0.807 |
| HAM-D | Combined sensory intervention | 5 days | 0.806 |
| HAM-D | Combined sensory intervention | 12 days | 0.826 |
| HAM-D | Combined sensory intervention | 21 days | 0.839 |
| HAM-D | Combined sensory intervention | 30 days | 0.821 |
| MADRS | Controls | Pre-infusion Baseline | 0.731 |
| MADRS | Controls | 24 hours | 0.765 |
| MADRS | Controls | 5 days | 0.778 |
| MADRS | Controls | 12 days | 0.781 |
| MADRS | Controls | 21 days | 0.779 |
| MADRS | Controls | 30 days | 0.778 |
| HAM-D | Controls | Pre-infusion Baseline | 0.811 |
| HAM-D | Controls | 24 hours | 0.814 |
| HAM-D | Controls | 5 days | 0.841 |
| HAM-D | Controls | 12 days | 0.830 |
| HAM-D | Controls | 21 days | 0.830 |
| HAM-D | Controls | 30 days | 0.824 |

**Supplementary Table S4.** Adverse events table for both study groups. Adverse events were collected using the Patient Rated Inventory of Side Effects (PRISE; (11)) and the Clinician-Administered Dissociative States Scale (CADSS; (12)). Open-ended questions were also used to inquire about any additional adverse events not captured on the PRISE. Adverse events were tallied for all symptoms that were reported as new or worsening from pre-infusion baseline with onset during the infusion or within the subsequent 24-hour period.

|  | | Combined Sensory Intervention Group | Controls |
| --- | --- | --- | --- |
|  | | Affected / n (%) | Affected / n (%) |
| Cardiac disorders | palpitations ^†^ | 4/22 (18.18%) | 2/21 (9.52%) |
| Gastrointestinal disorders | diarrhea ^†^ | 6/22 (27.27%) | 1/21 (4.76%) |
|  | dry mouth ^†^ | 7/22 (31.82%) | 9/21 (42.86%) |
|  | increased appetite ^†^ | 10/22 (45.45%) | 7/21 (33.33%) |
|  | nausea ^†^ | 4/22 (18.18%) | 6/21 (28.57%) |
| General disorders | decreased energy ^†^ | 3/22 (13.64%) | 7/21 (33.33%) |
|  | restlessness ^†^ | 5/22 (22.73%) | 3/21 (14.29%) |
|  | sweating ^†^ | 5/22 (22.73%) | 1/21 (4.76%) |
| Metabolism and nutrition disorders | increased weight ^†^ | 3/22 (13.64%) | 0/21 (0%) |
| Nervous system disorders | dizziness ^†^ | 9/22 (40.91%) | 14/21 (66.67%) |
|  | headache ^†^ | 4/22 (18.18%) | 5/21 (23.81%) |
|  | tremors ^†^ | 1/22 (4.55%) | 1/21 (4.76%) |
| Psychiatric disorders | anxiety ^†^ | 2/22 (9.09%) | 1/21 (4.76%) |
|  | difficulty sleeping: too little ^†^ | 4/22 (18.18%) | 2/21 (9.52%) |
|  | difficulty sleeping: too much ^†^ | 2/22 (9.09%) | 1/21 (4.76%) |
|  | dissociative symptoms ^†^ | 20/22 (90.91%) | 21/21 (100%) |
|  | emotional indifference ^†^ | 3/22 (13.64%) | 1/21 (4.76%) |
| Reproductive system and breast disorders | loss of sexual desire ^†^ | 3/22 (13.64%) | 0/21 (0%) |
|  | trouble achieving orgasm ^†^ | 2/22 (9.09%) | 1/21 (4.76%) |
| Vascular disorders | elevated blood pressure ^†^ | 1/22 (4.55%) | 0/21 (0%) |

**†** Indicates events were collected by systematic assessment.

**Supplementary Table S5-1.** MADRS and HAM-D total scores for combined sensory intervention group participants from pre-infusion baseline to each of the five post-infusion time points (24 hours, 5 days, 12 days, 21 days, and 30 days post-infusion) using Wilcoxon Signed Rank test (1) and paired samples *t*-test (2).

|  | Comparisons | Results* |
| --- | --- | --- |
| MADRS | 24 hours post-infusion vs pre-infusion | 1. Z = −3.92, p < 0.0001 2. t = −10.20, p < 0.0001 |
|  | 5 days post-infusion vs pre-infusion | 1. Z = −3.92, p < 0.0001 2. t = −7.32, p < 0.0001 |
|  | 12 days post-infusion vs pre-infusion | 1. Z = −3.98, p < 0.0001 2. t = −7.81, p < 0.0001 |
|  | 21 days post-infusion vs pre-infusion | 1. Z = −3.33, p = 0.001 2. t = −4.95, p < 0.0001 |
|  | 30 days post-infusion vs pre-infusion | 1. Z = −3.41, p = 0.001 2. t = −4.83, p < 0.0001 |
| HAM-D | 24 hours post-infusion vs pre-infusion | 1. Z = −3.92, p < 0.0001 2. t = −7.41, p < 0.0001 |
|  | 5 days post-infusion vs pre-infusion | 1. Z = −3.84, p < 0.0001 2. t = −6.25, p < 0.0001 |
|  | 12 days post-infusion vs pre-infusion | 1. Z = −3.88, p < 0.0001 2. t = −6.45, p < 0.0001 |
|  | 21 days post-infusion vs pre-infusion | 1. Z = −3.54, p < 0.0001 2. t = −4.73, p < 0.0001 |
|  | 30 days post-infusion vs pre-infusion | 1. Z = −3.25, p = 0.001 2. t = −4.47, p < 0.0001 |

* Results are reported for Wilcoxon Signed Rank test (1) and paired samples *t*-test (2). All results were controlled for Bonferroni corrections (adjusted alpha= 0.005).

MADRS: Montgomery-Asberg Depression Rating Scale; HAM-D: Hamilton Depression Rating Scale.

**Supplementary Table S5-2:** MADRS and HAM-D total scores for combined sensory intervention group participants from pre-infusion baseline to each of the five post-infusion time points (24 hours, 5 days, 12 days, 21 days, and 30 days post-infusion) using Generalized Estimation Equation (GEE).

|  | | Mean Difference | Std. Error | Bonferroni Sig.* | 95% Wald Confidence Interval for Difference | |
| --- | --- | --- | --- | --- | --- | --- |
|  |  |  |  |  | Lower | Upper |
| MADRS | Pre-infusion vs 24 hours post-infusion | 17.4785 | 1.57330 | 0.000 | 12.8605 | 22.0964 |
|  | Pre-infusion vs 5 days post- infusion | 14.8268 | 1.92721 | 0.000 | 9.1701 | 20.4836 |
|  | Pre-infusion vs 12 days post- infusion | 15.2078 | 1.98262 | 0.000 | 9.3884 | 21.0272 |
|  | Pre-infusion vs 21 days post- infusion | 11.7416 | 2.24018 | 0.000 | 5.1662 | 18.3170 |
|  | Pre-infusion vs 30 days post- infusion | 12.0864 | 2.28649 | 0.000 | 5.3751 | 18.7977 |
| HAM-D | Pre-infusion vs 24 hours post-infusion | 9.5545^a^ | 1.24349 | 0.000 | 5.9047 | 13.2044 |
|  | Pre-infusion vs 5 days post- infusion | 8.4545^a^ | 1.30258 | 0.000 | 4.6312 | 12.2779 |
|  | Pre-infusion vs 12 days post- infusion | 9.1212^a^ | 1.37886 | 0.000 | 5.0740 | 13.1685 |
|  | Pre-infusion vs 21 days post- infusion | 7.3493^a^ | 1.51488 | 0.000 | 2.9028 | 11.7957 |
|  | Pre-infusion vs 30 days post- infusion | 6.8045^a^ | 1.34847 | 0.000 | 2.8465 | 10.7626 |

* Results are reported for Generalized Estimation Equation (GEE). All results were adjusted for Bonferroni corrections and are significant at the 0.05 level.

MADRS: Montgomery-Asberg Depression Rating Scale; HAM-D: Hamilton Depression Rating Scale.

**Supplementary Table S6-1.** MADRS and HAM-D total scores for the control group from pre-infusion baseline to each of the five post-infusion time points (24 hours, 5 days, 12 days, 21 days, and 30 days post-infusion) using Wilcoxon Signed Rank test (1) and paired samples *t*-test (2).

* Results are reported for Wilcoxon Signed Rank test (1) and paired samples *t*-test (2). All results were controlled for Bonferroni corrections (adjusted alpha= 0.005).

MADRS: Montgomery-Asberg Depression Rating Scale; HAM-D: Hamilton Depression Rating Scale.

|  | Comparisons | Results* |
| --- | --- | --- |
| MADRS | 24 hours post-infusion vs pre-infusion | 1. Z = −3.92, p < 0.0001 2. t = −8.74, p < 0.0001 |
|  | 5 days post-infusion vs pre-infusion | 1. Z = −3.77, p < 0.0001 2. t = −6.81, p < 0.0001 |
|  | 12 days post-infusion vs pre-infusion | 1. Z = −3.43, p = 0.001 2. t = −5.21, p < 0.0001 |
|  | 21 days post-infusion vs pre-infusion | 1. Z = −3.56, p < 0.0001 2. t = −5.40, p < 0.0001 |
|  | 30 days post-infusion vs pre-infusion | 1. Z = −2.85, p = 0.004 2. t = −3.65, p = 0.002 |
| HAM-D | 24 hours post-infusion vs pre-infusion | 1. Z = −3.92, p < 0.0001 2. t = −8.75, p < 0.0001 |
|  | 5 days post-infusion vs pre-infusion | 1. Z = −3.82, p < 0.0001 2. t = −7.24, p < 0.0001 |
|  | 12 days post-infusion vs pre-infusion | 1. Z = −3.79, p < 0.0001 2. t = −6.20, p < 0.0001 |
|  | 21 days post-infusion vs pre-infusion | 1. Z = −3.60, p < 0.0001 2. t = −5.66, p < 0.0001 |
|  | 30 days post-infusion vs pre-infusion | 1. Z = −3.02, p = 0.003 2. t = −3.59, p = 0.002 |

**Supplementary Table S6-2.** MADRS and HAM-D total scores for the control group from pre-infusion baseline to each of the five post-infusion time points (24 hours, 5 days, 12 days, 21 days, and 30 days post-infusion) using Generalized Estimation Equation (GEE).

|  | | Mean Difference | Std. Error | Bonferroni Sig.* | 95% Wald Confidence Interval for Difference | |
| --- | --- | --- | --- | --- | --- | --- |
|  |  |  |  |  | Lower | Upper |
| MADRS | Pre-infusion vs 24 hours post-infusion | 16.9857a | 1.86594 | 0.000 | 11.5088 | 22.4626 |
|  | Pre-infusion vs 5 days post- infusion | 15.9857a | 2.24972 | 0.000 | 9.3823 | 22.5891 |
|  | Pre-infusion vs 12 days post- infusion | 13.0752a | 2.42062 | 0.000 | 5.9702 | 20.1802 |
|  | Pre-infusion vs 21 days post- infusion | 13.1857a | 2.33218 | 0.000 | 6.3403 | 20.0311 |
|  | Pre-infusion vs 30 days post- infusion | 9.1805a | 2.57374 | 0.005 | 1.6260 | 16.7349 |
| HAM-D | Pre-infusion vs 24 hours post-infusion | 10.6643a | 1.18221 | 0.000 | 7.1943 | 14.1343 |
|  | Pre-infusion vs 5 days post- infusion | 10.2143a | 1.36160 | 0.000 | 6.2177 | 14.2108 |
|  | Pre-infusion vs 12 days post- infusion | 9.0301a | 1.37553 | 0.000 | 4.9926 | 13.0675 |
|  | Pre-infusion vs 21 days post- infusion | 9.1643a | 1.53886 | 0.000 | 4.6474 | 13.6812 |
|  | Pre-infusion vs 30 days post- infusion | 6.2932a | 1.57371 | 0.000 | 1.6741 | 10.9124 |

* Results are reported for Generalized Estimation Equation (GEE). All results were adjusted for Bonferroni corrections and are significant at the 0.05 level.

MADRS: Montgomery-Asberg Depression Rating Scale; HAM-D: Hamilton Depression Rating Scale.

**Table S7.** Exploratory analysis of clinical outcomes between groups.

|  | Combined sensory intervention (n = 22*) | Control  (n = 21*) | Fisher’s Exact Test (p) | Chi-Square (χ², p) |
| --- | --- | --- | --- | --- |
| Responders (%) | 65% (13/20) | 70% (14/20) | 1.000 | 0.114  (p = 0.736) |
| Remitters (%) | 55% (11/20) | 45% (9/20) | 0.752 | 0.400  (p = 0.527) |
| Worsened (%) | 0% (0/20) | 0% (0/20) | — | — |

*One participant in the control group and two participants in the combined sensory intervention group were missing response or remission data at 24 hours post-ketamine.

44 participants were assessed in-person for eligibility

One participant enrolled and signed consent but never came for an infusion day

22 individuals were randomly assigned to receive mindfulness, music, and eye mask during ketamine infusion

All data from 22 individuals were used for quantitative analyses. Only 19 of the 22 participants in this group provided written responses and were thus included in the qualitative analysis.

All data from 21 individuals were used for quantitative analyses. Only 17 of the 21 participants in this group provided written responses and were thus included in the qualitative analysis.

43 randomized participants

21 individuals received ketamine alone (as controls)

**Supplementary Figure S1.** CONSORT diagram of study recruitment.


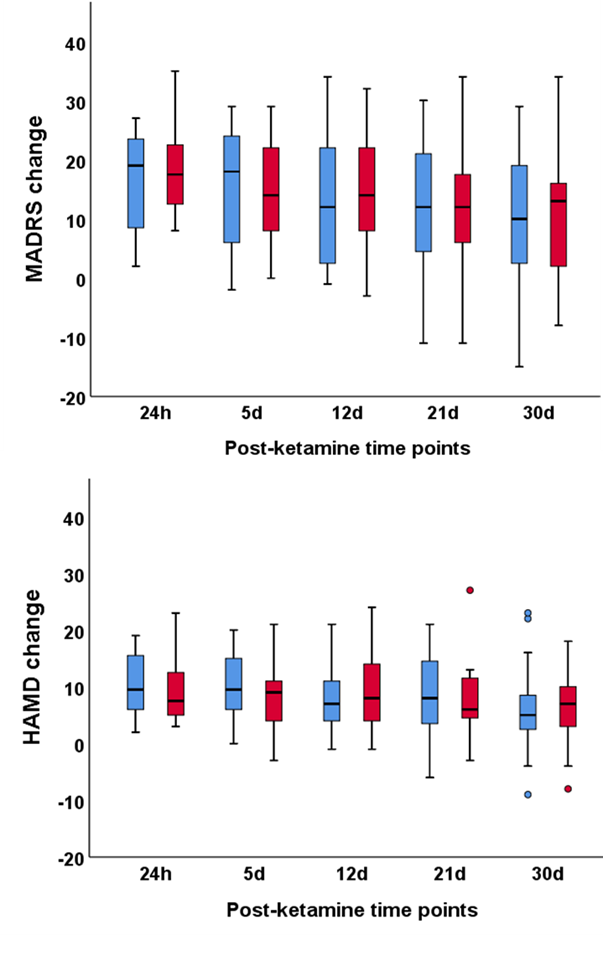


**Supplementary Figure S2**. Mongtomery-Asberg Depression Rating Scale (MADRS) and Hamilton Depression Rating Scale (HAM-D) change scores in participants who received the combined mindfulness, music, and eye mask interventions during ketamine infusion (red) compared to controls who received only ketamine without any additional interventions (blue) at five different time points post-ketamine infusion versus pre-infusion baseline. No significant differences were found between the two groups.

**Combined**

**Sensory**

**Intervention**

**Control**

**Combined**

**Sensory**

**Intervention**

**Control**


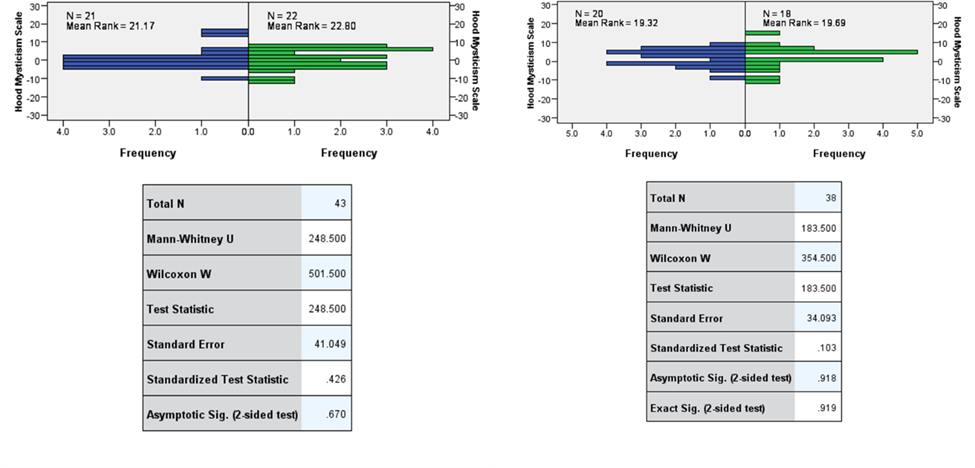


**Supplementary Figure S3**. Hood Mysticism Scale (HMS) scores for participants in the combined sensory intervention group (green) versus the control group (blue) at 40 minutes (left plot) and 24 hours (right plot) post-ketamine infusion. No significant differences were found between the two groups.

**Control**

**Combined**

**Sensory**

**Intervention**


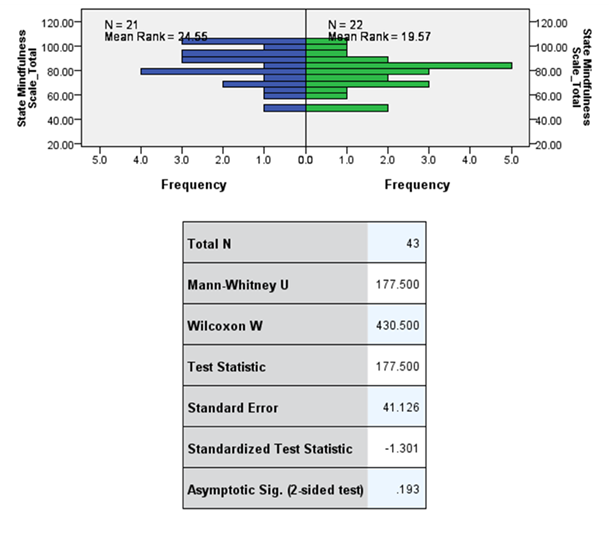


**Supplementary Figure S4**. State Mindfulness Scale (SMS) scores for participants in the combined sensory intervention group (green) versus the control group (blue) at 80 minutes post-ketamine infusion. No significant differences were found between the two groups.

**Control**

**Combined**

**Sensory**

**Intervention**


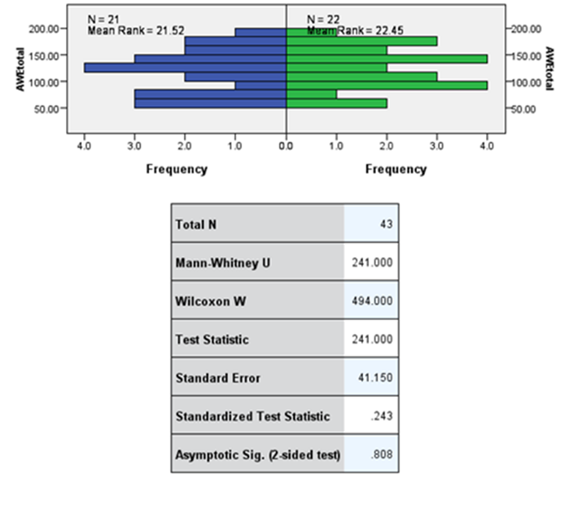


**Supplementary Figure S5**. Awe Experience Scale (AWE-S) scores for participants in the combined sensory intervention (green) versus the control group (blue) at 80 minutes post-ketamine infusion. No significant differences were found between the two groups.


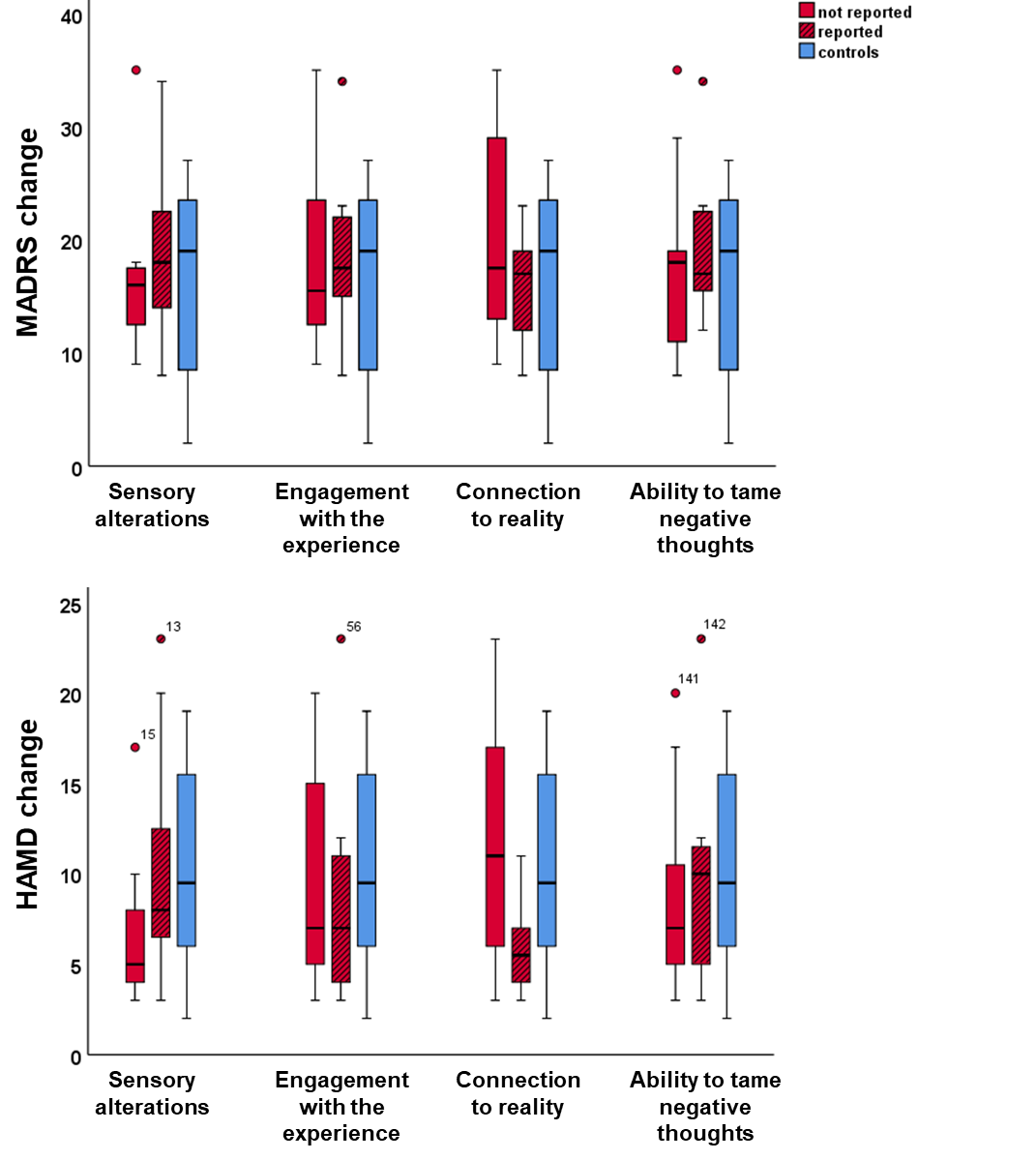


**Supplementary Figure S6**. Montgomery-Asberg Depression Rating Scale (MADRS) and Hamilton Depression Rating Scale (HAM-D) 24-hour change scores in participants who received the combined mindfulness, music, and eye mask intervention during ketamine infusion (red) compared to controls who received only ketamine without any additional interventions (blue). Participants who reported experiencing specific themes are shown in hatched red, while those who did not report these effects are shown in solid red. For exploratory purposes, these figures highlight potential relationships between qualitative experiences and quantitative mood changes across the four most frequently reported and non-reported subthemes in the combined sensory intervention group and controls.

Using non-parametric tests as an exploratory analysis (since the sample sizes in the reported and not-reported subgroups were too small), no significant differences were found across any themes or groups. However, in the combined sensory intervention group, participants who reported the subtheme "connection to reality through mindfulness, music, and eye mask" showed less change in HAM-D scores over 24 hours compared to both the non-reporting and control groups (p = 0.043 for both). While this significance disappeared after Bonferroni correction, these trends highlight potential areas for further investigation in future studies with larger sample sizes.

*Appendix A. Mindfulness Training Scripts*

**Introduction to Mindfulness**

- “Mindfulness” is a term used in many meditation practices and traditions. Have you heard of this term before? What does it mean to you?
- Although these techniques have been used for thousands of years, recent research has found that practicing mindfulness in order to develop this skill can help reduce depression symptoms, stress, anxiety, physical pain, and more.
- Jon Kabat-Zinn, an American researcher who teaches mindfulness to his patients, says that: “Mindfulness means paying attention in a particular way: on purpose, in the present moment, and nonjudgmentally.”
- Mindfulness starts when we recognize the tendency for us to be on “automatic pilot,” going through much of our day mindlessly, and then we decide to do our best to become aware of each moment. Asking ourselves simply, “what do I notice happening in this moment, right now?”
- Some people think that meditation is supposed to be about relaxation, but this is actually really challenging, hard work that takes practice. In order to build up this skill, it’s important not to critique or harshly judge oneself. Mindfulness means trying to accept whatever happens—which can include slipping into mind wandering or negative thought patterns. When this happens, just keep gently returning yourself to the present moment as soon as you can.
- Now we’ll ask you to listen to a pre-recorded, 20 minute guided meditation and follow the steps provided in order to practice mindfulness. Do you have any questions before we begin?
- Play “Complete Meditation Instructions” (19min) mp3 available at the following website: https://www.uclahealth.org/uclamindful/guided-meditations (https://creativecommons.org/licenses/by-nc-nd/4.0/)

**De-brief following guided meditation**

- What did you notice during the meditation exercise?
- What was challenging?
- What was different from your normal daily experience?

**Provide feedback and support, emphasizing:**

- non-judgmental stance
- acceptance and curiosity about whatever comes into awareness
- gently bringing yourself back to awareness of the present moment (for example, the breath, physical sensations throughout the body, sounds in the room), whenever you remember to do so

During the infusion today, we will ask you to keep practicing the mindfulness skill you have just practiced, by paying attention to the present moment, on purpose, and with a non-judgmental stance.

The infusion may provoke a wide range of experiences which may be quite unusual or different from your normal daily experience. These may include changes in the way your body feels, the way you experience the passage of time, the way things sound, thought patterns that are different from what you’re used to, or many other experiences. These experiences may be pleasant or unpleasant, and may fluctuate.

We ask that you simply do your best to notice and pay attention to whatever it is that is occurring, and work on being present in the moment. Whatever you do (or don’t) experience is perfectly ok.

Try to have a “Teflon mind,” letting experiences, feelings, and thoughts come into your mind and slip right out. See, but don’t evaluate. Push away nothing, and cling to nothing.

To help you work on staying present with your internal experience and to minimize distractions, we will provide a sleeping mask to cover your eyes and provide music to listen to throughout the infusion. Use these aids to help you delve deeply into the infusion experience. However, if you find they are making you uncomfortable in any way, you can choose to remove them at any time. I will be here in the room with you at all times.

Any questions?
